# Supplementary material for: The efficacy of coenzyme Q10 treatment in alleviating the symptoms of primary coenzyme Q10 deficiency: A systematic review
Source: J Cell Mol Med. 2022 Aug 19;26(17):4635–44. doi: 10.1111/jcmm.17488 (PMC9443948; doi:10.1111/jcmm.17488)
Supplement: Supplementary file 1 — Appendix S1 [file JCMM-26-4635-s001.pdf]

Supporting Information

The search query used in PUBMED

((((((((((((((COQ2[Title/Abstract]) OR (PDSS1[Title/Abstract])) OR (PDSS2[Title/Abstract])) OR (COQ3[Title/Abstract])) OR (COQ4[Title/Abstract])) OR (COQ[Title/Abstract])) OR (COQ6[Title/Abstract])) OR (COQ7[Title/Abstract])) OR (COQ8A[Title/Abstract])) OR (ADCK3[Title/Abstract])) OR (COQ8B[Title/Abstract])) OR (ADCK4[Title/Abstract])) OR (COQ9[Title/Abstract])) OR (COQ10[Title/Abstract])) AND (patient[Title/Abstract])) AND (primary[Title/Abstract])

Table S1 Primary CoQ10 deficiency patients identified by literature search.

S1.1 Primary CoQ_{10} deficiency-2 (COQ10D2; 614651) due to mutations in the *PDSS1* gene [# of patients: 2]

Gene [Patient ID]	# of patients (# of families)	Mutation	Level of CoQ_{10} (% of control)	Age at onset (sex) if known	Symptoms	Biochemical tests and muscle pathology	RCC enzymes	CoQ_{10} dose and responses	Age at last reported exam or death	Corresponding PI	Reference
**PDSS1 [1]**	2 (1)	D308E (HOM)	<5% (fibroblasts)	1-3 y/o (M)	encephalopathy, deafness, cardiac valvulopathy, livedo reticularis, mild mental retardation, macrocephaly, peripheral neuropathy, bulimia, obesity, and optic atrophy	mildly elevated blood lactate, mitochondrial aggregates in the muscle	CII+CIII ↓, G3PDH+CIII↓, CII+CIII/CS↓ (fibroblasts), CI+CIII/CI↓, CIII/CI+CIII ↑ (muscle mitochondria)	no data	22 years of age	Agnès Rötig, Hôpital Necker-Enfants Malades, France	(Mollet et al., 2007)
**PDSS1 [2]**		D308E (HOM)	<5% (fibroblasts)	2 y/o (F)	deafness, cardiac valvulopathy, obesity, macrocephaly, optic atrophy, peripheral neuropathy, livedo reticularis, mental retardation	mildly elevated blood lactate	no data	no data	14 years of age		

S1.2 Primary CoQ_{10} deficiency-3 (COQ10D3; 614652) due to mutations in the *PDSS2* gene [# of patients: 4]

Gene [Patient ID]	# of patients (# of families)	Mutation	Level of CoQ_{10} (% of control)	Age at onset (sex) if known	Symptoms	Biochemical tests and muscle pathology	RCC enzymes	CoQ_{10} dose and responses	Age at last reported exam or death	Corresponding PI	Reference
**PDSS2 [1]**	1 (1)	Q332X/ S382L (CH)	~ 14% (muscle) ~ 12% (fibroblasts)	3 m/o (M)	NS, hypotonia, Leigh syndrome, seizure	blood lactate↑, serum albumin↓, proteinuria mitochondrial aggregates in muscle	CII+CIII↓ (fibroblasts and muscle)	50mg/day beginning at age 3 months, no response *[NR]*	died at age of 8 months	Michio Hirano, Columbia University medical center, USA	(Lopez et al., 2006; Quinzii et al., 2008; Salviati et al., 2012)
**PDSS2 [2]**	1 (1)	S382L (HOM)	no data	1.9 y/o (M)	SRNS, cerebral palsy, intellectual disability	no data	no data	no data	no data	Friedhelm Hildebrandt,	

**PDSS2 [3]**	1 (1)	A384D (HOM)	no data	neonatal (M)	SRNS	no data	no data	no data	no data	Boston Children's Hospital, USA	(Sadowski et al., 2015)
**PDSS2 [4]**	1 (1)	H162R/ c.1042_1148-2816del (CH)	no data	neonatal (M)	NS, encephalomyopathy, hypertrophic cardiomyopathy, deafness, retinitis pigmentosa, global developmental delay	blood lactate↑, proteinuria	no data	20mg/kg/day, no response *[NR]*	died at age of 8 months (1 month after admission)	Béla Iványi, University of Szeged, Hungary	(Ivanyi et al., 2018)

S1.3 Primary CoQ$_{10}$ deficiency-1 (COQ10D1; 607426) due to mutations in the *COQ2* gene [# of patients: 25]

Gene [Patient ID]	# of patients (# of families)	Mutation	Level of CoQ$_{10}$ (% of control)[1]	Age at onset (sex) if known	Symptoms	Biochemical tests and muscle pathology	RCC enzymes	CoQ$_{10}$ dose and responses	Age at last reported exam or death	Corresponding PI	Reference
**COQ2 [1]**	1 (1)	R197H/ N228S (CH)	~ 36% (fibroblasts) <3% (kidney, muscle)	18 m/o (M)	SRNS	normal blood lactate	CII+CIII ↓ (muscle)	30mg/kg/day since age 21 months, response not described	29 months of age, ESRF at age 20 months	Francesca Diomedi-Camassei, Bambino Gesu` Children's Hospital, Italy	(Diomedi-Camassei et al., 2007; Quinzii et al., 2010)
**COQ2 [2]**	1 (1)	S146N (HOM)	~ 17% (fibroblasts) <3% (kidney, muscle)	neonatal (M)	glomerulonephritis, acute renal failure, seizure, epileptic encephalopathy	CSF lactate↑	CII+CIII↓ (muscle)	no data	died at 6 months of age	Francesca Diomedi-Camassei Bambino Gesu` Children's Hospital, Italy	(Diomedi-Camassei et al., 2007) (Bujan et al., 2014)
**COQ2 [3]**	2 (1)	Y297C (HOM)	~18% (fibroblasts) ~ 37.5% (muscle)	11 m/o (M)	infantile encephalomyopathy, SRNS/FSGS, hypotonia, optic atrophy, tremors, psychomotor regression	normal lactate levels, proteinuria; myofibers with excessive succinate dehydrogenase staining	CI+CIII ↓, CII+CIII ↓ (muscle), CII+CIII ↓ (fibroblasts)	30 mg/kg/day beginning at age 22months, neurologic picture improved, but no change in renal function *[NR]*	ESRF at age 18 months, kidney transplant at age of 3 years	Michio Hirano, Columbia University medical center, USA	(Diomedi-Camassei et al., 2007; Montini et al., 2008; Quinzii et al., 2006; Quinzii et al., 2008; Salviati et al., 2005)
**COQ2 [4]**		Y297C (HOM)	~ 17% (fibroblasts)	12 m/o (F)	NS/FSGS without any clinical signs of neurologic involvement.	proteinuria. hypoalbuminemia	CII+CIII ↓ (fibroblasts)	△30 mg/kg/day, there was no improvement during the first 2 weeks of treatment; an episode of acute renal failure required continuous hemofiltration for	5 years of age	Michio Hirano, Columbia University medical center, USA	(Diomedi-Camassei et al., 2007; Montini

								4 days. 20 days after the initiation of the treatment, recovery of renal function and a reduced level of proteinuria was observed. After 50 months of therapy, renal function remains normal, though proteinuria was still present (other medication: diuretics) *[NR]*			et al., 2008; Quinzii et al., 2006; Quinzii et al., 2008; Salviati et al., 2005)
**COQ2 [5]**	1 (1)	N401fsX 415 (HOM)	~ 24% (fibroblasts)	neonatal (F)	Infantile multiorgan failure (neurologic distress, liver failure, NS, anemia, pancytopenia, insulin-dependent diabetes, and seizures)	no data	CI+CIII ↓, CII+CIII ↓ (liver)	no data	died at 12 days	Agnès Rötig, Hôpital Necker-Enfants Malades, France	(Mollet et al., 2007; Quinzii and Hirano, 2010)
**COQ2 [6]**	1 (1)	L234fsX2 47/ N228S (CH)	no data	2 y/o (F)	SRNS	no data	no data	no data	4 years of age	Moin A. Saleem, Southmead Hospital, UK	(McCarth y et al., 2013)
**COQ2 [7]**	2 (1)	A302V (HOM)	~ 29.2% (muscle)	neonatal (F)	generalized edema, seizures, apnea, hypotonia, dystonic-hyper kinetic movement, feeding problems	blood lactate↑	CII+CIII↓ (muscle, fibroblasts)	no data	died at age of 5 months	R.J.T. Rodenburg, Radboud University, The Netherlands	(Jakobs et al., 2013)
**COQ2 [8]**		A302V (HOM)	~ 8.5% (fibroblasts), 3.4% (muscle)	neonatal (M)	generalized edema, seizures, apnea, hypotonia, dystonic-hyper kinetic movement, feeding problems	blood lactate↑, muscle histology is normal	CI↓, CIII↓, CII+CIII↓, ATP↓ (muscle), CII+CIII↓(fibroblast s)	no data	died at age of 6 months	R.J.T. Rodenburg, Radboud University, The Netherlands	(Jakobs et al., 2013; Ziosi et al., 2017)
**COQ2 [9]**	1 (1)	S109N (HOM)	~ 11.4% (fibroblasts)	neonatal (M)	peripheral hypertonia, cardiomyopathy, hypertrophic cardiomegaly, nephrotic syndrome	CSF lactate ↑, proteinuria	CII+CIII↓(kidney)	30mg/kg/day, no response *[NR]*	died at age of 5 months	Emmanuel Scalais, Centre Hospitalier de Luxembourg, Luxembourg	(Scalais et al., 2013; Ziosi et al., 2017)
**COQ2 [10]**	1 (1)	S146N/ R387X (CH)	no data	neonatal (F)	acidosis, hyperglycemia, cardiomegaly, respiratory distress, necrotizing enterocolitis, encephalopathy	blood lactate↑	CI↓, CII↓, CS↑ (muscle)	no data	died at age of 2 months	D. Dinwiddie, University Of New Mexico, USA	(Dinwidd ie et al., 2013)
**COQ2 [11]**	2 (1)	M128V-V393A (HOM)	<20% (brain)	68 y/o (F)	multiple system atrophy with predominant parkinsonism, retinitis pigmentosa	no data	no data	no data	died	Shoji Tsuji, University of Tokyo Japan	(Multiple -System Atrophy Research, 2013)
**COQ2 [12]**		M128V-V393A (HOM)	no data	62 y/o (M)	multiple system atrophy with predominant parkinsonism, ataxia, retinitis pigmentosa	no data	no data	no data	died		
**COQ2 [13]**	2 (1)	M387X/ V393A (CH)	no data	50 y/o (F)	multiple system atrophy of the cerebellar type	no data	no data	no data	no data		

**COQ2 [14]**		M387X/ V393A (CH)	no data	44 y/o (M)	multiple system atrophy of the cerebellar type	no data	no data	no data	no data		
**COQ2 [15]**	1 (1)	N228S (HOM)	no data	no data (M)	SRNS	no data	no data	no data	no data	Friedhelm Hildebrandt, Boston Children's Hospital, USA	Sadowski et al., 2015)
**COQ2 [16]**	1 (1)	R173H/ N228S (CH)	no data	2.5 y/o (M)	SRNS	no data	no data	no data	no data		
**COQ2 [17]**	1 (1)	N228S/ L286F (CH)	no data	1.3 y/o (F)	SRNS	no data	no data	no data	no data		
**COQ2 [18]**	1 (1)	Y297C (HOM)	no data	5 m/o (M)	SRNS	no data	no data	no data	no data		
**COQ2 [19]**	2 (1)	G390A (HOM)	no data	18 y/o (F)	SRNS/FSGS	dysmorphic mitochondria (kidney)	no data	treated, response not described	kidney transplant at age 20 years	L Gesualdo, University "Aldo Moro", Italy	(Gigante et al., 2017)
**COQ2 [20]**		G390A (HOM)	no data	16 y/o (F)	SRNS/FSGS	dysmorphic mitochondria (kidney)	no data	treated, response not described	kidney transplant at age 19 years		
**COQ2 [21]**	3 (1)	c.288dup C/ R126G (CH)	no data	25 y/o (M)	diffuse glomerulosclerosis, end-stage nephropathy, retinopathy	no data	no data	30 mg/kg/day for 6 months, no ERG improvement, but best corrected visual acuity and areas of retinal atrophy on autofluorescence were noted to be stable on treatment *[NR]*	25 years of age	Stephen H. Tsang, Columbia University Irving Medical Center, USA	(Abdelhakim et al., 2020)
**COQ2 [22]**		c.288dup C/ R126G (CH)	no data	21 y/o (M)	mesangial sclerosis, end-stage nephropathy, retinopathy, lymphoma	no data	no data		32 years of age, kidney transplant at age 5 years		
**COQ2 [23]**		c.288dup C/ R126G (CH)	no data	23 y/o (F)	retinopathy, end-stage nephropathy	no data	no data		28 years of age, kidney transplant at age 10 years		
**COQ2 [24]**	2 (1)	Y353C/ T325A (CH)	no data	2 y/o (M)	SRNS/FSGS	no data	no data	no data	died of ESRF at 5 years of age	Liangzhong Sun, Southern Medical University, China	(Li et al., 2021)
**COQ2 [25]**		Y353C/ T325A (CH)	no data	7 m/o (F)	SRNS	no data	no data	30 mg/kg/ day beginning at age 11 months, urinary protein decreased with the increasing dose of CoQ_{10}, now on the dosage of 600mg/day *[Obj.]*	normal growth at 4 years old		

S1.4 Primary CoQ_{10} deficiency-7 (COQ10D7; 616276) due to mutations in the *COQ4* gene [# of patients: 32]

Gene [Patient ID]	# of patients (# of families)	Mutation	Level of CoQ_{10} (% of control)[1]	Age at onset (sex) if known	Symptoms	Biochemical tests and muscle pathology	RCC enzymes	CoQ_{10} dose and response	Age at last reported exam or death	Corresponding PI	References
**COQ4 [1]**	1 (1)	mono-allelic deletion (CH)	~ 43% (fibroblasts)	neonatal (M)	dysmorphic features, mental retardation, encephalomyopathy	blood lactate in normal range, increased SDH staining in the muscle	CII+CIII↓ (fibroblasts)	30 mg/kg/day, improvement in physical status and social function. Conditions worsened (weakness and diffuse myalgia) after formulation change and dosage reduction to 2mg/kg/day. Remission of symptoms within a week after reverting back to the original dosage. Then switched to 15mg/kg/day of ubiquinol *[Obj.]*	3 years of age	Plácido Navas, Universidad Pablo de Olavide, Spain	(Salviati et al., 2012)
**COQ4 [2]**	1 (1)	R145G (HOM)	~ 41-54% (fibroblasts), ~ 23% (muscle)	neonatal (M)	hypotonia, areflexia, acrocyanosis, bradycardia, respiratory insufficiency, left ventricular hypoplasia	blood lactate↑, blood creatine kinase↑	CI+CIII↓, CII+CIII↓, CI↓ (autoptic muscle), CII+CIII↓ (fibroblasts)	not treated	died at 4 hours after birth	Holger Prokisch, Technische Universität München, Germany	(Brea-Calvo et al., 2015; Ziosi et al., 2017)
**COQ4 [3]**	1 (1)	R141X/ G240C (CH)	no data	neonatal (F)	respiratory failure, lactic acidosis, cardiomyopathy, heart failure	urinary and plasmatic amino acids, organic acids, and acylcarnitine are normal	CI ↓, CII ↓, CIII ↓, CIV ↓, CI+CIII ↓ (autoptic muscle)	not treated	died at 4 hours after birth	Holger Prokisch, Technische Universität München, Germany	(Brea-Calvo et al., 2015)
**COQ4 [4]**	2 (1)	L52S/ T174del (CH)	~ 2% (muscle)	neonatal (F)	distal arthrogryposis, respiratory distress, encephalopathy, multiorgan failure	blood lactate↑	CIV↓, CII+CIII↓ (autoptic muscle)	not treated	died at 3 days after birth		
**COQ4 [5]**		L52S/ T174del (CH)	~ 3% (muscle)	neonatal (F)	respiratory distress, encephalopathy	blood lactate↑, amino acids in plasma↑, analysis of urinary organic acids showed mitochondrial dysfunctional excretion pattern	CIII↑, CIV↑ (autoptic muscle)	not treated	died at 2 days after birth		
**COQ4 [6]**	1 (1)	P64S (HOM)	~ 63% (muscle)	10 m/o (M)	motor deterioration, ataxia, epileptic seizures, swallowing impairment, progressive scoliosis, cognitive deterioration	blood tests excluded liver and kidney involvement and showed no lactic acidosis	CI↓, CIII↓, CI+CIII ↓ (muscle)	treated, response not described	17 years of age		

**COQ4 [7]**	2 (1)	L82Q/ R158Q (CH)	~ 16% (muscle)	neonatal (F)	seizures, severe lactic and respiratory acidosis, heart failure	blood and CSF lactate↑, plasma alanine↑, increased mitochondrial size in the muscle	CII+CIII↓ (muscle)	△20 mg/kg/day beginning at the first day of life, which resulted in normalization of lactate and improvement in cardiac function. Nevertheless, the patient continued exhibiting intermittent episodes of lactic acidemia and cardiac decompensation until death (other medications: thiamine, riboflavin, hydroxocobalamin, biotin) *[NR]*	died at 2 months of age	Marwan Shinawi, Washington University School of Medicine, USA	(Chung et al., 2015)
**COQ4 [8]**		not tested	no data	neonatal (F)	respiratory distress, metabolic acidosis, apnoeic/gasping episode	no data	no data	not treated	died at 36 hours of life		
**COQ4 [9]**	1 (1)	R240C (HOM)	no data	neonatal (F)	hypotonia, cardiomyopathy, cerebellar and brainstem hypoplasia	lactic and pyruvic aciduria	normal ETC complex activities (muscle)	not treated	died at 4 days of life		
**COQ4 [10]**	2 (1)	R66Q/ D68H (CH)	no data	neonatal (F)	seizure, respiratory distress, intractable epilepsy, hypotonia, feeding difficulties, cardiomyopathy, and global developmental delay	blood lactate in normal range, a slight increase of CSF lactate	no data	no data	died at age of 19 months		
**COQ4 [11]**		not tested	no data	neonatal (F)	hypotonia, metabolic acidosis,	blood lactate↑, urinary malate and fumarate↑	no data	no data	died at age of 10 weeks		
**COQ4 [12]**	1 (1)	R240C (HOM)	no data	neonatal (F)	poor/absent reflexes, cardiac hypertrophy, left hip dysplasia, hypotonia, episodes of apnea and bradycardia	normal lactate, pyruvate, ammonia, creatine phosphokinase, acylcarnitine and plasma amino acids, increased lactic acid, 2-ketoglutaric acid, fumarate and 2-hydroxyglutaric acid in urine, CSF lactate↑	no data	△15 mg/kg/day beginning at age 1 month, no response [other medications: pyridoxal phosphate, folinic acid, and riboflavin] *[NR]*	died at 7 weeks old		
**COQ4 [13]**	1 (1)	V8AfsX19/D111Y + P119L (CH)	~ 21% (muscle) ~ 34% (fibroblasts)	neonatal (M)	seizure, ventricular hypertrophy, bilateral hearing loss, hypotonia	blood lactate↑	CI+CIII↓, CII+CIII↓ (fibroblasts)	no data	died at 4 months old	Ali B. Naini, Columbia University Medical Center, USA	(Sondheimer et al., 2017)
**COQ4 [14]**	2 (1)	T77I (HOM)	no data	4 y/o (M)	tremors, dysarthria, seizure, spastic tetraparesis and ataxia	no data	no data	1000mg/day beginning at age 13, the 6 min walk test was	15 years of age	Jan-Maarten Cobben,	(Bosch et al., 2018)

								stable over the period of a year *[NR]*		University of Amsterdam, the Netherlands	
**COQ4 [15]**		T77I (HOM)	~ 22% (fibroblasts)	9 y/o (F)	seizure, dysarthria, spastic tetraparesis, ataxia	general laboratory tests were normal	no data	1000mg/day beginning at age 11, the 6 min walk test was stable over a year, developed a second stroke-like episode at age 14 *[NR]*	14 years of age		
**COQ4 [16]**	2 (1)	G124S (HOM)	no data	neonatal (M)	motor deterioration, weak responsiveness, hearing impairment, dystonia, seizure, tachycardia, respiratory distress	blood lactate↑, glucose↑, blood ammonia↑, no evidence of renal impairment	no data	no data	died at 5.6 months	Qiwei Guo, Xiamen University, China	(Lu et al., 2019)
**COQ4 [17]**		G124S (HOM)	~ 50% (fibroblasts)	neonatal (F)	motor deterioration, weak responsiveness, dystonia, nystagmus, respiratory distress, seizure	blood lactate↑, glucose↑, blood ammonia↑	CII+CIII↓ (fibroblasts)	△50 mg/kg/day, improvement in seizure, screaming, and respiratory distress, no improvement in nystagmus, dystonia, psychomotor development, and ambulation *[NR]*	1 year of age		
**COQ4 [18]**	1 (1)	P193S/ R240C (CH)	~ 95% (fibroblasts)	2.5 y/o (M)	developmental delay, hypotonia, sialorrhea, spasticity, ataxia	no data	no change of CII+CIII activity (fibroblasts)	30 mg/kg/day of ubiquinol, improvement in neuromuscular symptoms after 2 months, further improvement of motor skills in the following months, but speech delay and cognitive impairment persisted *[Subj.]*	2.7 years of age	Maria Marchese, IRCCS Fondazione Stella Maris, Italy	(Mero et al., 2021)
**COQ4 [19]**	1 (1)	G95D/ R102H (CH)	~ 98% (fibroblasts)	5 y/o (F)	cognitive impairment, dysmetria, spastic ataxia, seizure	no data	no change of CII+CIII activity (fibroblasts), normal RCC activities (muscle)	100mg/kg/day of ubiquinol, no response after 6 months (as assessed by the SARA scale) *[NR]*	19 years of age		
**COQ4 [20]**	2 (1)	G55V (HOM)	normal range (blood)	8 y/o (M)	ataxia, spasticity, epilepsy, cognitive deterioration, dysarthria, dysmetria and dysdiadochokinesia	no data	no data	2000 mg/day, improvement of SARA score, dysarthria is persistent *[obj.]*	27 years of age	Margit Burmeister, University of Michigan, USA	(Caglayan et al., 2019)
**COQ4 [21]**			normal range (blood)	8 y/o (F)	dysarthria, spastic ataxia, epilepsy, cognitive deterioration, dysmetria, dysdiadochokinesia	no data	no data	Treated, dose not described, improvement of SARA score, gait difficulty and dysarthria are persistent *[obj.]*	28 years of age		
**COQ4 [22]**	1 (1)	E161D (HET)	~ 25% (fibroblasts)	4 y/o (F)	mental retardation, rhabdomyolysis	muscle damage, rhabdomyolysis, disorganized intermyofibrillar pattern, SDH and COX staining↓ in the muscle	CI+CIII↓, CII+CIII ↓ (fibroblasts)	no data	4 years of age	Pablo Menendez, CIBERONC, Spain	(Romero-Moya et al., 2017)

**COQ4 [23]**	1 (1)	G124S/ c.402+1 G>C (CH)	low (fibroblasts)	neonatal (M)	encephalopathy, cardiomyopathy, visual and hearing impairment, respiratory failure, apnea, developmental delay	blood lactate↑	CII+CIII↓ (fibroblasts)	40 mg/kg/day beginning at 5 months of age, poor response *[NR]*	died at 8 months of age	Brian Hon-Yin Chung, Hong Kong Children’s Hospital, China	(Yu et al., 2019)
**COQ4 [24]**	1 (1)	G124S/ c.402+1 G>C (CH)	no data	neonatal (M)	cardiomyopathy, respiratory distress, metabolic acidosis	blood lactate and alanine↑	no data	△15 mg/kg/day, no response [other medication: carnitine] *[NR]*	died at 2.5 days of age		
**COQ4 [25]**	1 (1)	G124S (HOM)	no data	neonatal (F)	cardiomyopathy, seizure, developmental delay	blood lactate↑, hyperammonemia	no data	△ treated, dose not described, cardiac function improved gradually and normalized after 10 days [other medication: intravenous immunoglobulin] *[NR]*	9 months of age		
**COQ4 [26]**	2 (1)	G124S/ c.402+1 G>C (CH)	no data	neonatal (F)	seizure, apnea, encephalopathy, cardiomyopathy	blood lactate↑	no data	started at the age of 4 years and 5 months, dose not described, no response observed after 1 month of treatment *[NR]*	4.5 years of age		
**COQ4 [27]**			no data	2 m/o (F)	seizure, respiratory distress, cardiomegaly	blood lactate↑	no data	started at 1 year of age, dose not described, no response, passed away 1 month later *[NR]*	died at 1.1 years of age		
**COQ4 [28]**	1 (1)	W184R/ c.402+1 G>C (CH)	low (fibroblasts)	8 m/o (M)	microcephaly, developmental delay, dystonia, visual impairment, oro-motor dysfunction	blood lactate and alanine↑	CII+CIII↓ (fibroblasts)	dose not described, no response *[NR]*	3.6 years of age		
**COQ4 [28]**	1 (1)	G124S (HOM)	low (fibroblasts)	infancy (F)	visual impairment, dystonia, spasticity, developmental delay	blood lactate↑	CII+CIII↓ (fibroblasts)	since age of 2 , dose not described, no response *[NR]*	died at 3.5 years of age		
**COQ4 [30]**	1 (1)	G124V/ G124S (CH)	low (fibroblasts)	infancy (F)	encephalopathy, dystonia, spasticity, developmental delay, visual impairment, seizure	blood lactate and alanine↑	CII+CIII↓ (fibroblasts)	△beginning at 9 months of age, dose not described, subjective improvement in response [other medication: levetiracetam] *[NR]*	3.3 years of age		
**COQ4 [31]**	1 (1)	G124S (HOM)	low (fibroblasts)	2 m/o (M)	encephalopathy, spasms, seizure, development delay	blood lactate↑	CII+CIII↓ (fibroblasts)	beginning at 7 years of age, dose not described, response not described	8 years of age		
**COQ4 [32]**	2 (1)	G124S (HOM)	no data	2 m/o (F)	hypotonia, developmental delay, bilateral cortical blinding, seizure, cardiomyopathy	blood lactate↑	no data	30mg/kg/day beginning at 11 months of age, some improvement in seizure control and development *[Subj.]*	1.5 years of age		

S1.5 Primary CoQ_{10} deficiency-9 (COQ10D9; 619028) due to mutations in the *COQ5* gene [# of patients: 3]

Gene [Patient ID]	# of patients (# of families)	Mutation	Level of CoQ_{10} (% of control)[1]	Age at onset (sex) if known	Symptoms	Biochemical tests and muscle pathology	RCC enzymes	CoQ_{10} dose and response	Age at last reported exam or death	Corresponding PI	Reference
**COQ5 [1]**	3 (1)	biallelic duplication of last 4 exons	~ 57% (muscle) ~ 50% (leukocytes)	childhood (F)	ataxia, dysarthria, seizures, cognitive disability, behavioral problems, epilepsy, myoclonus, dysarthric cerebellar speech, dysmetria, mild tremors and mild lower limb spasticity	liver and renal function tests, carnitine and acyl-carnitine, lactate, pyruvate, ammonia, blood amino acid profile, urine for protein and organic acids, were within normal limits.	CII + III↓ (fibroblasts)	dose not described, improvement of ICARS scoring after 3 months, the patient appeared to have a quicker response rate during conversation and better alertness *[Obj.]*	17 years of age	Yair Anikster, Bruria Ben-Zeev, Edmond and Lily Safra Children's Hospital, Israel	(Malicdan et al., 2018)
**COQ5 [2]**			~ 66% (leukocytes)	childhood (F)	mild static gait ataxia, mild dysarthria, mild dysmetria and oculomotor apraxia, and horizontal nystagmus	no data	no data	dose not described, improvement of ICARS scoring after 3 months, the patient appeared to have a quicker response rate during conversation and better alertness *[Obj.]*	22 years of age		
**COQ5 [3]**			~ 60% (leukocytes)	childhood (F)	mild motor delay, mild learning difficulties, mild cerebellar ataxia, mild cerebellar dysarthria and horizontal nystagmus	no data	no data	dose not described, improvement of ICARS scoring after 3 months, the patient appeared to have a quicker response rate during conversation and better alertness *[Obj.]*	14 years of age		

S1.6 Primary CoQ_{10} deficiency-6 (COQ10D6; 614650) due to mutations in the *COQ6* gene [# of patients: 28]

Gene [Patient ID]	# of patients (# of families)	Mutation	Level of CoQ_{10} (% of control)[1]	Age at onset (sex) if known	Symptoms	Biochemical tests and muscle pathology	RCC enzymes	CoQ_{10} dose and response	Age at last reported exam or death	Corresponding PI	Reference
**COQ6 [1]**	4 (1)	G255R (HOM)	no data	6.4 y/o	SRNS, SND	no data	no data	not treated	6.5 years of age; ESRF at age of 9.3 years	Friedhelm Hildebrandt, University of Michigan, USA	(Heeringa et al., 2011)
**COQ6 [2]**		G255R (HOM)	no data	0.3 y/o	SRNS, SND	no data	no data	not treated	died at age of 17.5 years		
**COQ6 [3]**		G255R (HOM)	no data	1.2 y/o	SRNS, SND, ataxia	no data	no data	not treated	died at age of 6.5 years		

**COQ6 [4]**		not tested	no data	<1 y/o	SRNS, congenital SND	no data	no data	not treated	died at 5 years old		
**COQ6 [5]**	3 (1)	G255R (HOM)	no data	0.3 y/o	SRNS, seizure	no data	no data	not treated	died, age of death not described		
**COQ6 [6]**		G255R (HOM)	no data	0.3 y/o	SRNS, SND, facial dysmorphism	no data	no data	100mg/day, improvement of SND *[NR]*	ESRF at age of 0.4 year		
**COQ6 [7]**		G255R (HOM)	no data	0.2 y/o	SRNS, SND, bilateral nephrolithiasis	no data	no data	△30mg/kg/day beginning at 2 months of age (together with enalapril), a decrease of proteinuria, SND and severe growth retardation were noted at 10 months of age	15 months of age		
**COQ6 [8]**	2 (1)	A353D (HOM)	no data	6.0 y/o	SRNS, SND	no data	no data	not treated	ESFR at age of 6.5 years		
**COQ6 [9]**		A353D (HOM)	no data	2.5 y/o	SRNS, SND	no data	no data	beginning at age 5.5 years, dose not described, decrease of proteinuria but no hearing improvement, reoccurrence of proteinuria after temporary cessation of CoQ_{10} treatment and it decreased again after the treatment resumed *[Obj.]*	6 years of age		
**COQ6 [10]**	1 (1)	A353D (HOM)	no data	2.5 y/o	SRNS, seizure, white matter abnormalities	no data	no data	not treated	died, age of death unknown		
**COQ6 [11]**	1 (1)	W447X/ Q461fsX4 78 (CH)	no data	3.0 y/o	SRNS, SND	no data	no data	not treated	3 years of age		
**COQ6 [12]**	1 (1)	R162X/ ?	no data	no data	cyclosporine A-dependent NS	no data	no data	no data	no data		
**COQ6 [13]**	1 (1)	W188X/ ?	no data	no data	diffuse mesangial sclerosis	no data	no data	no data	no data		
**COQ6 [14]**	1 (1)	A353D (HOM)	no data	4 y/o (M)	SRNS	no data	no data	no data	no data	Friedhelm Hildebrandt, Boston Children's Hospital, USA	(Sadowski et al., 2015)
**COQ6 [15]**	1 (1)	A353D (HOM)	no data	3.2 y/o (M)	SRNS	no data	no data	no data	no data		
**COQ6 [16]**	1 (1)	D385A/ Y412C (CH)	no data	4.5 y/o (M)	SRNS	no data	no data	no data	no data		
**COQ6 [17]**	1 (1)	R360W/ c.804del C (CH)	no data	2 y/o (F)	steroid-resistant glomerulopathy, poor growth	proteinuria (mostly during respiratory tract infection)	no data	30 mg/kg/day, remission of glomerulopathy after 1 month of treatment, growth acceleration after 12 months and a reduction of respiratory airway infections *[NR]*	4 years of age	Małgorzata Stańczyk University of Lodz, Poland	(Koyun et al., 2019; Stanczyk et al., 2018)

**COQ6 [18]**	1 (1)	P261L (HOM)	no data	0.8 y/o (M)	SRNS	no data	no data	treated, response not described	4 years of age	L Gesualdo, University of Bari Aldo Moro, Italy	(Gigante et al., 2017)
**COQ6 [19]**	1 (1)	K64del/ P261L (CH)	no data	3.8 y/o (M)	steroid-resistant FSGS, mild muscle weakness in the lower extremities	no data	no data	no data	no data	Hae Il Cheong, Seoul National University Hospital, South Korea	(Park et al., 2017a)
**COQ6 [20]**	1 (1)	K64del/ Q229P (CH)	no data	1.8 y/o (F)	SR-FSGS, exotropia with nystagmus	no data	no data	no data	no data		
**COQ6 [21]**	1 (1)	K64del/ P261L (CH)	no data	3.9 y/o (F)	SR-FSGS	no data	no data	no data	no data		
**COQ6 [22]**	1 (1)	K64del/ P261L (CH)	no data	2.7 y/o (F)	SR-FSGS	no data	no data	no data	no data		
**COQ6 [23]**	1 (1)	K64del/ P261L (CH)	no data	1.3 y/o (F)	SR-FSGS, optic nerve atrophy	no data	no data	no data	no data		
**COQ6 [24]**	1 (1)	K64del/ P261L (CH)	no data	2.1 y/o (M)	SR-FSGS, mild muscle weakness in the lower extremities	no data	no data	no data	no data		
**COQ6 [25]**	2 (1)	A353D (HOM)	no data	5 y/o (M)	SRNS, SND, optic atrophy	no data	normal CI+CIII and CII+CIII activities (muscle)	△15mg/kg/day of idebenone beginning at age of 17 years after the onset of optical symptoms, an improvement in the visual acuity after 2 months of treatment. After 13 months of treatment, the optical examination was stable, but the patient did not recover normal vision, still exhibiting persistent optic atrophy. After 3 years of treatment, minimal optic atrophy was reported. No change of the deafness status since treatment initiation. [other medications: immunosuppressive treatment]	Age of 18 years, kidney transplant at age 6	Justine Perrin, Hôpital Sainte-Musse, France	(Justine Perrin et al., 2020)
**COQ6 [26]**		A353D (HOM)	no data	4 y/o (M)	SRNS, SND	no data	no data	△10mg/kg/day of idebenone since age 7, after 13 months of treatment, hearing loss was not changed and renal involvement remained stable with only Enalapril, demonstrated by negative proteinuria.	8 years of age		

**COQ6 [27]**	2 (1)	Y83X/Q461 (CH)	no data	4 m/o (F)	seizure, growth retardation, proteinuria, atrial septal defect, and pulmonary hypertension	blood lactate↑, lipids ↑, albumin↓, urine organic acid ↑	no data	not treated	died at age of 5 months	Lizhen Wang, Wenzhou Medical University	(Wang et al., 2021)
**COQ6 [28]**			no data	3 m/o (M)	proteinuria, growth retardation, and muscle hypotonia	blood lactate↑, triglyceride ↑, albumin↓, edema	no data	not treated	died at age of 4 months		

S1.7 Primary CoQ_{10} deficiency-8 (COQ10D8; 616733) due to mutations in the *COQ7* gene [# of patients: 6]

Gene [Patient ID]	# of patients (# of families)	Mutation	Level of CoQ_{10} (% of control)[1]	Age at onset (sex) if known	Symptoms	Biochemical tests and muscle pathology	RCC enzymes	CoQ_{10} dose and response	Age at last reported exam or death	Corresponding PI	References
**COQ7 [1]**	1 (1)	V141E (HOM)	~ 10% (fibroblasts, muscle)	neonatal (M)	muscular hypotonia, developmental retardation, learning disabilities, hearing impairment, visual dysfunction, not able to sit and walk independently	blood and CSF lactate↑/small fiber size, no abnormal mitochondrial structure observed in the muscle	CI+CIII ↓, CII+CIII ↓ (fibroblasts), CI+CIII ↓, CI ↓ (muscle)	initially treated with idebenone, switched to COQ_{10} after the diagnosis of a primary CoQ_{10} deficiency (around age of 10 years), dosage unknown, stalling the regression and significantly reducing the pain were noted *[NR]*	9 years of age	Anna Wredenberg, Karolinska Institutet, Sweden	(Freyer et al., 2015)
**COQ7 [2]**	1 (1)	L111P (HOM)	~ 70% (fibroblasts)	14 m/o (F)	spasticity, muscle wasting, inability to walk without support	CSF lactate↑	no data	22.8 mg/kg/day, no response after 3 months of treatment *[NR]*	6 years of age	Siegfried Hekimi, McGill University, Canada	(Wang et al., 2017b)
**COQ7 [3]**	1 (1)	K200Ifs X56/ R107W (CH)	~ 12% (fibroblasts)	neonatal (M)	cardiomyopathy, growth retardation, hypotonia, ptosis, visual impairment, hearing impairment, muscle weakness, infantile spasms	blood lactate and alanine↑, urinary lactate↑, pyruvate↑, and 3-hydroxybutyrate↑, dicarboxylic aciduria, excretions of Kreb cycle intermediates↑	no data	Beginning at 2 months of age, and the dose was increased to 20 mg/kg/day at 12 months of life, the patient died around the same time *[NR]*	1 year of age	Cheuk-Wing Fung and Brian H.-Y. Chung, Queen Mary Hospital, China	(Kwong et al, 2019)
**COQ7 [4]**	1 (1)	R54Q (HOM)	~ 55 % (fibroblasts)	15 m/o (M)	hypotonia, difficulty walking, motor developmental delay, ataxia, and spasticity	no data	no data	not treated	6 years of age	Evren Gumus, Mugla Sitki Kocman University, Turkey	https://doi.org/10.1016/j.ymgmr.2022.100877
**COQ7 [5]**	2 (1)	I66N/Y149C (CH)	no data	Pediatric (unknown)	axonal neuropathy, mild neurodegenerative disorder	no data	no data	no data	no data	Hubert Smeets, Maastricht University, Netherlands	(Theunissen et al., 2018)
**COQ7 [6]**											

S1.8 Primary CoQ_{10} deficiency-4 (COQ10D4; 612016) due to mutations in the *COQ8A/ADCK3* gene [# of patients: 112]

Gene [Patient ID]	# of patients (# of families)	Mutation	Level of CoQ_{10} (% of control)[1]	Age at onset (sex) if known	Symptoms	Biochemical tests and muscle pathology	RCC enzymes	CoQ_{10} dose and response[2]	Age at last reported exam or death	Corresponding PI	References
**COQ8A [1]**	2 (1)	R213W/ G272V (CH)	~ 29% (muscle)	18 m/o (F)	hypotonia, *talus valgus*, developmental delay, seizure, ataxia, epilepsia partialis continua	blood and CSF lactate in normal range	CI↑, CII↑, CIII↑, CIV↑ (muscle)	20 mg/kg/day (350mg/day) for 8 years, no response *[NR]*	21 years of age	Agnès Rötig, Hôpital Necker-Enfants Malades, France	(Mignot et al., 2013; Mollet et al., 2008)
**COQ8A [2]**		R213W/ G272V (CH)	no data	2 y/o (F)	hypotonia, seizure, ataxia, developmental delay	blood and CSF lactate in normal range	no data	350mg/day for 13 months, no response *[NR]*	15 years of age		
**COQ8A [3]**	1 (1)	E551K (HOM)	~ 8% (muscle), normal range (fibroblasts)	18 m/o (M)	cerebella ataxia, strabismus, muscle weakness, trunk hypotonia, tonic seizure	blood lactate↑, no ragged-red fibers in the muscle but mitochondrial accumulation and lipid droplets in 10%–20% of the fibers	CI+CIII↓(muscle)	5mg/kg/day from age 3 years, 10mg/kg/day from age 4 to 7, no response; followed by 10mg/kg/day of idebenone for 7 months which worsened the patient's conditions *[NR]*	16 years of age		(Mollet et al., 2008)
**COQ8A [4]**	1 (1)	G272D/ Q605GfsX 125 (CH)	< 5% (muscle), normal range (fibroblasts)	3 y/o (F)	exercise intolerance, muscle weakness, cerebellar syndromes, seizure	blood lactate↑, mitochondrial myopathy in the muscle	CI↑, CII↑, CIII↑, CIV↑, CS↑, CI+CIII↓, CII+CIII↓ (muscle)	△6 mg/kg/day (750mg/day) of CoQ_{10} and L-carnitine were initiated at age 5, improved exercise tolerance and fewer vomiting episodes were noted after 3 months of therapy. CoQ_{10} was replaced with idebenone (5mg/kg/day) at the age of 9 years, and within the following 4 months, severe exercise intolerance reappeared with numerous episodes of vomiting. Reverting to CoQ_{10} treatment resulted in returns to the previous clinical status within 3 months. *[Obj.]*	20 years of age	Anne Lombès, Hospitalier Pitié-Salpêtrière, France	(Aure et al., 2004; Mignot et al., 2013; Mollet et al., 2008)
**COQ8A [5]**	4 (1)	c.1398+2T →C (D420WfsX 40, I67AfsX22) (HOM)	no data	11 y/o (M)	cerebellar ataxia	no data	no data	not treated	42 years of age	Michel Koenig, Hôpitaux Universitaires de Strasbourg, France	(Lagier-Tourenne et al., 2008; Quinzii et al., 2010)
**COQ8A [6]**		c.1398+2T →C (D420WfsX 40, I67AfsX22) (HOM)	no data	4 y/o (M)	cerebellar ataxia, exercise intolerance	blood lactate↑	no data	not treated	38 years of age		(Lagier-Tourenne et al., 2008)

**COQ8A [7]**		c.1398+2T →C (D420WfsX 40, I67AfsX22) (HOM)	normal range (fibroblasts)	7 y/o (M)	Cerebellar ataxia, exercise intolerance	blood lactate↑	CI+CIII↓ (fibroblasts)	not treated	36 years of age		(Lagier-Tourenne et al., 2008)
**COQ8A [8]**		c.1398+2T →C (D420WfsX 40, I67AfsX22) (HOM)	no data	8 y/o (F)	Cerebellar ataxia, exercise intolerance	blood lactate↑	no data	not treated	29 years of age		(Lagier-Tourenne et al., 2008)
**COQ8A [9]**	1 (1)	Q167LfsX 36 (HOM)	~ 64% (fibroblasts)	4 y/o (M)	cerebellar ataxia, mild mental retardation	blood lactate in normal range	CI+CIII ↓ , CII+CIII ↓ (fibroblasts)	not treated	18 years of age		(Lagier-Tourenne et al., 2008; Quinzii et al., 2010)
**COQ8A [10]**	1 (1)	Y514C/ T584del (CH)	~ 51% (fibroblasts), ~ 46% (muscle)	5 y/o (M)	cerebellar ataxia, gynecomastia, feet and thumbs in dystonic position	blood lactate in normal range	CI+CIII ↓ , CII+CIII ↓ (fibroblasts)	60 -700 mg/day over 8 years, the patient reported mild subjective improvement, and stabilization of the cerebellar ataxia was observed on examination	17 years of age		(Lagier-Tourenne et al., 2008; Lamperti et al., 2003; Quinzii et al., 2010)
**COQ8A [11]**	1 (1)	K314_Q360 del/ G549S (CH)	no data	3 y/o (F)	cerebellar ataxia, mild hearing loss	blood lactate in normal range	no data	not treated	30 years of age		(Lagier-Tourenne et al., 2008)
**COQ8A [12]**	3 (1)	R348X (HOM)	no data	3 y/o (M)	cerebellar ataxia, exercise intolerance, myoclonus, tremor, dystonic posture	no data	no data	no data	31 years of age	Hubert Smeets, Maastricht University, The Netherlands	(Gerards et al., 2010)
**COQ8A [13]**		R348X (HOM)	no data	9 y/o (M)	cerebellar ataxia, cognitive impairment, speech and coordination difficulties, exercise intolerance	no data	no data	no data	26 years of age		
**COQ8A [14]**		R348X (HOM)	no data	3 y/o (M)	cerebellar ataxia, epilepsy, exercise intolerance, vision impairment	no data	no data	no data	25 years of age		
**COQ8A [15]**	2 (1)	R348X/ L379X (CH)	no data	2 y/o (F)	ataxia, tremor, dysarthric and monotonous speech, exercise intolerance, slight spasticity	unremarkable muscle morphology	no data	no data	26 years of age		
**COQ8A [16]**		R348X/ L379X (CH)	no data	infancy (M)	cerebellar ataxia, dysarthria	unremarkable muscle morphology	CII+CIII↓ (muscle)	no data	21 years of age		
**COQ8A [17]**	1 (1)	R348X (HOM)	<14.5% (muscle)	6 y/o (F)	seizure, ataxia, cerebellar atrophy, a mild cognitive delay	laboratory tests, including creatine kinase, and	CII+CIII ↓ (muscle)	10mg/kg/day initiated at the age of 8 years, within 6 months improvement of	17 years of age	Enrico Bertini,	(Terracciano et al., 2012)

						metabolic investigations, including transferrin isofocusing, serum lactate, serum and urine organic acids, were unremarkable		ataxia was observed, but after 5 years of treatment, MRI showed increased cerebellar atrophy		Bambino Gesù Children's Hospital, Italy	
**COQ8A [18]**	2 (1)	T584delACC/ P502R (CH)	no data	2 y/o (F)	cerebellar ataxia, dysarthria, nystagmus, cognitive decline, psychiatric disorder	metabolic evaluation and muscle morphology were unremarkable	CI+CIII↓, CIV↓(muscle)	20mg/kg/day initiated at age 5, partial improvement in motor skills, balance, and strength; after 6 years, treatment was discontinued, and the patient's condition deteriorated. *[Obj.]*	20 years of age	Dorit Lev, Wolfson Medical Center, Israel	(Blumkin et al., 2014)
**COQ8A [19]**		T584delACC/ P502R (CH)	no data	childhood (F)	mild dysfluent speech and clumsiness, cerebellar atrophy, mild dysarthria	no data	no data	treated, dosage and response not described	32 years of age		
**COQ8A [20]**	1 (1)	S616LfsX114/ R301Q (CH)	~ 45% (plasma)	9 y/o (M)	exercise intolerance, cerebellar ataxia, tremors, dysautonomia	blood lactate↑, the remaining blood tests, including liver function and serum creatine kinase were all normal	no data	120mg/day, self-reported fatigue and exercise tolerance improved after 2 weeks of therapy. After 2 years of therapy, ataxia and head tremor diminished and SARA total score improved. When the treatment was stopped for a month, the patient's condition deteriorated, rendering him to resume taking CoQ_{10}. *[Obj.]*	35 years of age	Dantao Peng, China-Japan Friendship Hospital, China	(Zhang et al., 2020)
**COQ8A [21]**	1 (1)	R271C/ A304T (CH)	normal range (muscle)	15 y/o (F)	cerebellar ataxia, tremors	no data	COX↓(muscle)	300 mg/day, no response after 6 months *[NR]*	46 years of age	Rita Horvath, Newcastle University, UK	(Horvath et al., 2012)
**COQ8A [22]**	1 (1)	A304V (HOM)	~ 8% (muscle)	27 y/o (F)	cerebellar ataxia, upper-limb myoclonus, seizure, dysmetria, cataract	no data	CI↓, CIV↓, COX↓, lipid↑ (muscle)	300 mg/day, no response after 6 months *[NR]*	50 years of age		
**COQ8A [23]**	1 (1)	R299W (HOM)	no data	1 y/o (F)	cerebellar ataxia, seizure, mental retardation, unable to walk by 12 years	no data	no data	200 mg/day, no response within 2 months *[NR]*	18 years of age		
**COQ8A [24]**	1 (1)	Y429C/?	~ 22% (muscle)	1.5-2 y/o (F)	ataxia, muscle weakness, cognitive impairment, horizontal nystagmus, bilateral dysmetria, tremors	no data	CI↓, CIV↓, CII+CIII↓, COX↓, lipid ↑ (muscle)	200 mg/day, no response within 2 months *[NR]*	20 years of age		
**COQ8A [25]**	2 (1)	S616LfsX114 (HOM)	~ 35% (fibroblasts)	10 y/o (F)	cerebellar ataxia, myoclonus, slurred speech, wheelchair-dependent by 30 years of age	no data	CI↓, CII+CIII↓ (fibroblasts)	400mg/day, improvement in myoclonic symptoms, speech quality (after 3 months), and ataxia with a reduction in	35 years of age	Henry Houlden, National Hospital for Neurology and	(Liu et al., 2014)

								SARA (after 6 months) *[Obj.]*		Neurosurgery, UK	
**COQ8A [26]**		S616LfsX 114 (HOM)	no data	14 y/o (M)	cerebellar ataxia, myoclonus, tremors, dysarthric speech	no data	no data	200mg/day, improvement in speech and fatigue after 3 months of treatment	32 years of age		
**COQ8A [27]**	1 (1)	R301W/ c.1399-3_ 1408del (CH)	low (muscle)	11 y/o (M)	reduced dexterity, dysarthria, hypometric saccades, scanning speech, and dystonic posturing, tremors, ataxia	no data	no data	800mg/day, a resolution of tremors and improvement of limb and truncal dystonia after 9 months of treatment *[Subj.]*	25 years of age		
**COQ8A [28]**	1 (1)	T584del/ T511M (CH)	low (muscle)	10 y/o (F)	ataxia, tremors, dysarthria, appendicular dysmetria, truncal instability, titubation, wheelchair-dependent by 53 years of age	no data	no data	800mg/day, improvement of ataxia overall with a reduction in SARA score, able to work independently, after 9 months of therapy. *[Obj.]*	54 years of age	Renato Puppi Munhoz, University of Toronto, Canada	(Chang et al., 2018)
**COQ8A [29]**	1 (1)	D305Y (HOM)	low (muscle)	5 y/o (M)	developmental delay, intellectual disability, ataxia, isolated pan-cerebellar features including head titubation, dysmetria, dysdiadochokinesia	no data	normal range of activities of CI and CII, CS and COX (muscle)	800mg/day, inconsistent use for 2 years, no response *[NR]*	33 years of age		
**COQ8A [30]**	1 (1)	T445RfsX 52 (HOM)	no data	no data (F)	ataxia, seizure, developmental delay, strabismus	no data	no data	no data	15 years of age		
**COQ8A [31]**	1 (1)	T511M (HOM)	no data	no data (F)	ataxia, developmental delay	no data	no data	no data	20 years of age		
**COQ8A [32]**	1 (1)	R348X/ 2A>G [p?] (CH)	no data	no data (F)	ataxia, dysarthria	no data	no data	no data	45 years of age	Miao Sun, The University of Chicago, USA	(Sun et al., 2019)
**COQ8A [33]**	1 (1)	R271C/ R334W (CH)	no data	25 y/o (M)	ataxia, dystonia, myoclonus, tremors, seizure	no data	no data	no data	31 years of age		
**COQ8A [34]**	1 (1)	E551K/ R301W (CH)	no data	no data (F)	ataxia, seizure	no data	no data	no data	33 years of age		
**COQ8A [35]**	1 (1)	R301W/ R410Q (CH)	no data	no data (F)	ataxia, developmental delay	no data	no data	no data	8 years of age		
**COQ8A [36]**	1 (1)	N148X/ A338T (CH)	no data	12 y/o (F)	cerebellar ataxia, tremors, focal dystonia	blood lactate in normal range	no data	not treated	35 years of age		
**COQ8A [37]**	1 (1)	A42fs/ Q50X (CH)	no data	no data (F)	cerebellar ataxia, dystonic tremor	no data	no data	not treated	38 years of age	Matthis Synofzik University of Tübingen, Germany	(Traschutz et al., 2020)
**COQ8A [38]**	1 (1)	A339T/ Y361 (CH)	no data	42 y/o (M)	cerebellar ataxia, stroke-like episode, muscle weakness, hearing loss	blood lactate in normal range, no ragged red fibers in the muscle	activities in normal range	dosage not described, no response *[NR]*	45 years of age		

**COQ8A [39]**	2 (1)	A337T (HOM)	no data	6 y/o (M)	cerebellar ataxia, dystonia, tremor,	blood lactate in normal range	no data	600mg/day, no response *[NR]*	12 years of age		
**COQ8A [40]**		A337T (HOM)	no data	2 y/o (M)	ataxia, impairment of speech	no data	no data	not treated	6 years of age		
**COQ8A [41]**	1 (1)	A338V (HOM)	no data	13 y/o (F)	cerebellar ataxia, muscle weakness, myoclonus, tremor, dysarthria	blood lactate in normal range	no data	dosage not described, improved tremors	18 years of age		
**COQ8A [42]**	2 (1)	V83fs (HOM)	no data	8 y/o (F)	cerebellar ataxia, tremors	blood lactate ↑	no data	1250mg/day, improved tremors	37 years of age		
**COQ8A [43]**		V83fs (HOM)	no data	16 y/o (M)	cerebellar ataxia, dysarthria, tremors	blood lactate in normal range	no data	1250mg/day, response not described	25 years of age		
**COQ8A [44]**	1 (1)	T584del/ A338T (CH)	~ 15% (muscle)	6 y/o (F)	ataxia, pan-cerebellar atrophy	mild mitochondrial myopathy with ragged red fibers and COX- fibers (muscle)	normal RC enzyme activities (muscle)	100mg/day, response not described	69 years of age		
**COQ8A [45]**	1 (1)	E481X (HOM)	no data	1 y/o (F)	ataxia, motor retardation, cognitive impairment, tremors	blood lactate in normal range	no data	200mg/day, no initial apparent effect but after stop: fatigue and falls; improvement of muscle weakness with reintroduction of CoQ_{10}	16 years of age		
**COQ8A [46]**	1 (1)	Q167LfsX 36/ R348X (CH)	no data	1 y/o (M)	ataxia	no data	no data	not treated	17 years of age		
**COQ8A [47]**	1 (1)	R271C/ T487R (CH)	no data	6 y/o (F)	ataxia, seizure, stroke-like episodes	unremarkable muscle histology	normal range (muscle)	not treated	26 years of age		
**COQ8A [48]**	1 (1)	c.589-3C>G/ G615D (CH)	no data	2 y/o (F)	ataxia, hypotonia	blood lactate in normal range	no data	10 mg/kg/day, improvement in stability	9 years of age		
**COQ8A [49]**	1 (1)	R348X (HOM)	no data	25 y/o (M)	ataxia, tremors, spasticity	no data	no data	not treated	53 years of age		
**COQ8A [50]**	1 (1)	c.589-3C>G/ R301W (CH)	no data	2 y/o (F)	ataxia, seizure	no data	no data	10 mg/kg/day, improved balance	13 years of age		
**COQ8A [51]**	2 (1)	R301W/ E446AfsX 33 (CH)	low (muscle)	3 y/o (M)	ataxia	mild muscle histology changes	mild changes (muscle)	10 mg/kg/day, no response *[NR]*	10 years of age		
**COQ8A [52]**		R301W/ E446AfsX 33 (CH)	no data	2 y/o (M)	ataxia, developmental retardation	no data	no data	10 mg/kg/day, no response *[NR]*	7 years of age		

**COQ8A [53]**	1 (1)	R348X (HOM)	low (muscle)	10 y/o (F)	epilepsy, ataxia	blood lactate in normal range	normal range (muscle)	600mg/day, no response *[NR]*	24 years of age		
**COQ8A [54]**	1 (1)	R301W (HOM)	low (muscle)	8 y/o (F)	ataxia, seizure, cardiomyopathy	blood lactate in normal range	normal range (muscle)	400mg/day, no response *[NR]*	death at 17 years of age		
**COQ8A [55]**	1 (1)	E568X (HOM)	no data	6 y/o (F)	spastic hypertonia, ataxia	no data	no data	300mg/day since 5 years old, more energetic, mentally quicker	69 years of age		
**COQ8A [56]**	2 (1)	M555I (HOM)	no data	11 y/o (F)	ataxia, exercise intolerance, cognitive complaints	no data	no data	not treated	46 years of age		
**COQ8A [57]**		M555I (HOM)	no data	1 y/o (F)	ataxia, memory/concentration difficulties	unremarkable muscle histology	no data	not treated	40 years of age		
**COQ8A [58]**	1 (1)	Q167Lfs (HOM)	no data	1 y/o (M)	developmental delay, hypomimia, learning difficulties, ataxia	blood lactate↑	no data	not treated	19 years of age		
**COQ8A [59]**	1 (1)	O207L (HOM)	no data	11 y/o (F)	ataxia, tremors, myoclonus	no data	no data	not treated	19 years of age		
**COQ8A [60]**	1 (1)	H85AfsX42 (HOM)	no data	3 y/o (M)	ataxia	blood lactate in normal range	no data	400 - 1200 mg/day, response not described	9 years of age		
**COQ8A [61]**	1 (1)	L275RfsX16/ L402P (CH)	no data	6 y/o (F)	ataxia, choreiform dyskinesia, tremors	blood lactate in normal range	no data	not treated	16 years of age		
**COQ8A [62]**	2 (1)	C268R (HOM)	no data	6 y/o (F)	ataxia, epilepsy	no data	no data	not treated	23 years of age		
**COQ8A [63]**		C268R (HOM)	no data	2 y/o (F)	developmental delay, epilepsy, cerebellar atrophy	no data	no data	not treated	21 years of age		
**COQ8A [64]**	1 (1)	M555I (HOM)	no data	1 y/o (M)	hearing loss, tremors, cerebellar atrophy	no data	no data	not treated	59 years of age		
**COQ8A [65]**	1 (1)	R301W/ M555I (CH)	no data	4 y/o (F)	mental and motor retardation, ataxia, tremors, seizure	blood lactate in normal range	no data	not treated	58 years of age		
**COQ8A [66]**	1 (1)	G342W (HOM)	no data	10 y/o (M)	ataxia, tremors	no data	no data	not treated	78 years of age		
**COQ8A [67]**	2 (1)	R213G (HOM)	no data	childhood (M)	ataxia, speech and swallowing difficulties, leg cramps	no data	no data	not treated	63 years of age		
**COQ8A [68]**		R213G (HOM)	no data	20 y/o (F)	ataxia, seizure, speech and swallowing difficulties	blood lactate in normal range	no data	not treated	58 years of age		
**COQ8A [69]**	1 (1)	I4K/ R512W (CH)	no data	2 y/o (M)	mental retardation, ataxia	no data	no data	not treated	18 years of age		
**COQ8A [70]**	1 (1)	L453RfsX24/ E568X (CH)	no data	13 y/o (F)	tremors, incoordination	blood lactate in normal range	no data	not treated	37 years of age		
**COQ8A [71]**	1 (1)	L453RfsX24/ E568X (CH)	no data	22 y/o (F)	ataxia, bipolar disorder, impulsive behavior	no data	no data	not treated	71 years of age		

**COQ8A [72]**	2 (1)	T445fs (HOM)	no data	7 y/o (F)	mild ataxia, mild dysarthria	no data	no data	not treated	41 years of age		
**COQ8A [73]**		T445fs (HOM)	no data	6 y/o (M)	mild ataxia, mild dysarthria tremors, speech disorder	mild denervation in muscle	no data	not treated	37 years of age		
**COQ8A [74]**	2 (1)	G615D (HOM)	no data	childhood (M)	ataxia, dysmetria, seizure	blood lactate in normal range	no data	135mg/day of idebenone for 9 months, response not described	29 years of age	Mathieu Anheim, Hôpital de la Salpêtrière, France	(Mignot et al., 2013)
**COQ8A [75]**		G615D (HOM)	no data	7 y/o (F)	ataxia. dysmetria	no data	no data	135mg/day of idebenone, response not described	24 years of age		
**COQ8A [76]**	1 (1)	del exons 3-15/ F508S (CH)	no data	6 y/o (M)	ataxia. dysmetria, myoclonus	no data	no data	300mg/day for 15 months, improvement in movement disorder and SARA score *[Obj.]*	17 years of age		
**COQ8A [77]**	1 (1)	R299W/ L453RfsX 24 (CH)	normal range (fibroblasts)	15 y/o (M)	ataxia, seizure, myoclonus, dysmetria	no data	no data	300mg/day for 8 months, improvement in movement disorder *[Subj.]*	44 years of age		
**COQ8A [78]**	2 (1)	R299W/ R410X (CH)	no data	4 y/o (F)	ataxia, dysmetria, seizure	no data	no data	300mg/day for1 month, withdrawn, reversible side effect of treatment (anorexia) *[NR]*	38 years of age		
**COQ8A [79]**		R299W/ R410X (CH)	no data	4 y/o (M)	ataxia, dysmetria, seizure	blood lactate in normal range	no data	300mg/day for1 month, withdrawn, reversible side effect of treatment (diarrhea) *[NR]*	34 years of age		
**COQ8A [80]**	1 (1)	R271C (HOM)	low (plasma)	1.5 y/o (F)	ataxia, seizure, dystonia, chorea, dysmetria, myoclonus, spasticity	blood lactate in normal range	CII+CIII↓ (muscle)	30 mg/kg/day for 3 years, no response *[NR]*	5 years of age		
**COQ8A [81]**	2 (1)	L197VfsX 20 (HOM)	no data	19 y/o (F)	ataxia. dysmetria	blood lactate in normal range	no data	1200mg/day for 1 year no response *[NR]*	34 years of age		
**COQ8A [82]**		L197VfsX 20 (HOM)	no data	19 y/o (F)	ataxia. dysmetria, seizure	blood lactate in normal range	no data	1200mg/day for 1 year, no response *[NR]*	31 years of age		
**COQ8A [83]**	1 (1)	Q360_Y36 1insX (HOM)	no data	2 y/o (F)	ataxia. Dysmetria, tremors	blood lactate in normal range	no data	800mg/day for 1 year, no response *[NR]*	15 years of age		
**COQ8A [84]**	1 (1)	R299W (HOM)	~ 10-24% (muscle)	7 y/o (F)	ataxia, seizure, tremor	unremarkable muscle biopsy	no data	900mg/day for 6 months, no response *[NR]*	35 years of age	L. A. Bindof, University of Bergen, Norway	(Hikmat et al., 2016)
**COQ8A [85]**	2 (1)	R299W/ F578V (CH)	~ 34-60% (muscle)	7 y/o (M)	ataxia, seizure, dysmetria, tremors, dysarthria, dysdiadochokinesia	unremarkable muscle biopsy	no data	600mg/day since the age of 33, improvement in balance and coordination (reported by the patient) and a reduction of SARA score *[Obj.]*	34 years of age		
**COQ8A [86]**		R299W/ F578V (CH)	no data	3 y/o (F)	dysarthria, ataxia, epilepsy, cognitive impairment, tremors	no data	no data	no data	died at age of 22		
**COQ8A [87]**	1 (1)	R299W (HOM)	no data	2 y/o (F)	ataxia, epilepsy, seizure, feeding difficulties	unremarkable muscle biopsy	no data	1000mg/day of deoxyubiquinone (probably	22 years of age		

								ubiquinol) since age of 18, no response *[NR]*			
**COQ8A [88]**	1 (1)	R299W/ E551K (CH)	no data	5 y/o (M)	dysarthria, ataxia, seizure, delayed growth	blood lactate in normal range	no data	no data	18 years of age		
**COQ8A [89]**	1 (1)	A304V (HOM)	no data	10 y/o (M)	mild developmental delay, ataxia	no data	no data	no data	41 years of age	Mathieu Anheim, Hôpital de Hautepierre, France	(Mallaret et al., 2016)
**COQ8A [90]**	1 (1)	R299W/ L453RfsX 24 (CH)	no data	15 y/o (M)	mild developmental delay, ataxia	no data	no data	no data	46 years of age		
**COQ8A [91]**	1 (1)	27.6 kb deletion of 1q42.3 involving exons 1 and 2 (HOM)	~ 34% (muscle), normal range (fibroblasts)	13 y/o (F)	ataxia, tremors, hand bradykinesia, subtle and variable speech dysfluency	no data	CI+CIII↓, CII+CIII ↓, CS ↓ (muscle)	△ Tremor improved on trihexyphenidyl/clonazepam combination therapy before ubiquinol supplementation which was initiated at age 19 years. Ubiquinol dosage was not described. After two years of ubiquinol and high-dose vitamin B-complex treatments, tremor was stable, and the patient was able to tandem walk normally. She had marked bradykinesia though.	25 years of age		
**COQ8A [92]**	1 (1)	G615D/ L197VfsX 20 (CH)	no data	7 y/o (F)	tremors, ataxia, dysmetria, difficulty writing and hand clumsiness	metabolic work-up, including, plasma amino acids, plasma acyl-carnitine profile, urinary organic acids, lactate, was normal	no data	800mg/day initiated at the age of 8.5, clinical stabilization was reported after the treatment	10 years of age	Jennifer Friedman, Rady Children's Hospital, USA	(Galosi et al., 2019)
**COQ8A [93]**	1 (1)	R348X (HOM)	no data	25 y/o (M)	ataxia, tremors, writing difficulties	Blood creatine kinase and cholesterol ↑, the remaining blood biochemistry including urinary organic acids was normal	no data	no data	54 years of age		
**COQ8A [94]**	1 (1)	R301W/ E446AfsX 33 (CH)	no data	3 y/o (M)	ataxia, speech difficulties, seizure, tremors, dystonia	no data	no data	10 mg/kg/day, initiated at age 10, but has been taken only intermittently, response not described	11 years of age		
**COQ8A [95]**	2 (1)	L277P/ c.1506+1 G>A	low (muscle)	childhood (F)	ataxia, dysmetria, hypotonia	The metabolic workup, including	CII+CIII↓ (muscle)	20 mg/kg/day, improvement in an ataxia assessment score at 1-year follow-up *[Obj.]*	7.8 years of age	R. G. Snell,	(Jacobsen et al., 2018)

		(CH)	normal range (plasma)			plasma cholesterol, plasma albumin, plasma and urinary amino acids, urinary organic acids, and CSF lactate was normal. Mitochondria in the muscle were unremarkable.				The University of Auckland, New Zealand	
**COQ8A [96]**		L277P/ c.1506+1 G>A (CH)	normal range (plasma)	childhood (F)	ataxia		CII+CIII↓ (muscle)	20 mg/kg/day, minimal improvement in an ataxia assessment score at 1-year follow-up *[NR]*	2.2 years of age		
**COQ8A [97]**	1 (1)	c.655+1G >A/ A339T (CH)	no data	3 y/o (F)	exercise intolerance, dysarthria, seizure, stroke-like episodes, ataxia, homonymous hemianopsia, dysarthria	blood creatinine kinase↑, blood lactate↑, CI↓ and ragged red fibers in the muscle	no data	400mg/day, response not described	18 years of age	Young-Mock Lee, University College of Medicine, Korea	https://doi.org/10.26815/acn.2020.00276
**COQ8A [98]**	1 (1)	R410X (HOM)	no data	2 y/o (M)	ataxia, dysarthria	no data	no data	no data	7 years of age	Zhi-Ying Wu, Zhejiang University, China	(Cheng et al., 2021)
**COQ8A [99]**	1 (1)	R277H/ R301W (CH)	no data	9 y/o (M)	ataxia, dysarthria, cognition impairment	no data	no data	no data	11 years of age		
**COQ8A [100]**	1 (1)	R598H/ S616fs (CH)	no data	14 y/o (M)	ataxia, head and hands shaking	no data	no data	no data	17 years of age		
**COQ8A [101]**	1 (1)	S616fs (HOM)	no data	24 y/o (M)	ataxia, head and hands shaking, dysphagia	no data	no data	no data	26 years of age		
**COQ8A [102]**	1 (1)	L320fs (HOM)	no data	32 y/o (F)	ataxia, dysarthria, cognition impairment	no data	no data	no data	52 years of age		
**COQ8A [103]**	2 (1)	c.656-1G>T (HOM)	no data	20 y/o (F)	ataxia, writer's cramp	blood lactate in normal range	no data	60mg/day of ubiquinol, initiated at 20 years old, stopped after only 2 months due to incompliance, no response *[NR]*	45 years of age	Elisabetta Indelicato, University of Innsbruck, Austria	(Amprosi et al., 2021)
**COQ8A [104]**		c.656-1G>T (HOM)	no data	7 y/o (M)	ataxia, writer's cramp	no data	no data	60mg/day of ubiquinol, initiated at 25 years old, due to adverse event (frequent headache); switched to 5mg/kg/day of CoQ_{10}; no response at 1-year follow-up *[NR]*	28 years of age		
**COQ8A [105]**	1 (1)	A339T (HOM)	no data	14 m/o (F)	hypotonia, developmental delay, ataxia, glaucoma, dysmorphic features	serum creatine kinase↑; other investigations, including urinary organic acids and blood lactate were unremarkable. ragged-red fibers in the muscle.	CII+CIII↓, CS↑ (muscle)	100mg/day, response not described	6 years of age	Robert W. Taylor, Newcastle University, UK	(Cotta et al., 2020)

**COQ8A [106]**	1 (1)	Q343_V344delinsHM/ G244_Q284del (CH)	<2% (muscle), normal range (blood white cells)	2 y/o (F)	speech difficulties, ataxia, tremors, hypotonia, seizure, hypertension, exercise intolerance	blood lactate↑, blood alanine↑, TCA metabolites in urine↑, ragged-red fibers in the muscle.	CII+CIII↓, CS↑ (muscle)	no data	16 years of age		
**COQ8A [107]**	2 (1)	R301W/ E446AfsX33 (CH)	no data	3 y/o (M)	ataxia, tremors, epilepsy, mild intellectual retardation	no data	no data	15 mg/kg/day for 6 months, no improvement in motor performance (Timed 25-foot walk test, SARA) *[NR]*	10 years of age	Tommaso Schirinzi, Bambino Gesù Hospital, Italy	(Schirinzi et al., 2019)
**COQ8A [108]**		R301W/ E446AfsX33 (CH)	no data	3 y/o (M)	ataxia, mild intellectual retardation	no data	no data		7 years of age		
**COQ8A [109]**	1 (1)	G615D/ L197VfsX20 (CH)	no data	6 y/o (F)	ataxia, tremors	no data	no data	15 mg/kg/day for 1 year, improvement in Timed 25-foot walk but no significant change in SARA, gait analysis parameters and 6 min walking test *[NR]*	8 years of age		
**COQ8A [110]**	1 (1)	R301W/ c.589-3C > G (splice) (CH)	no data	2 y/o (F)	epilepsy, mild intellectual retardation	no data	no data		13 years of age		
**COQ8A [111]**	1 (1)	G27C (HOM)	no data	2 y/o (F)	seizure, developmental regression, hypothyroidism, mitral regurgitation, mitral valve prolapse, cerebellar atrophy, and epilepsia partialis continua	no abnormality in hematological and biochemical laboratory tests	no data	treated with CoQ_{10} after 11 years of age, dosage unknown, no effect on seizure frequency *[NR]*	11 years of age	Morteza Heidari, Tehran University of Medical Sciences, Iran	(Ashrafi et al., 2022)
**COQ8A [112]**	1 (1)	L609V (HET)	moderate deficiency in fibroblasts and muscle	(F)	ataxia	no data	CII+CIII↓ (muscle)	30mg/kg/day from 8 years old, a reduction in ICARS after years of treatment *[Obj.]*	10 years of age	Rafael Artuch, Hospital Sant Joan de Dèu, Spain	(Pineda et al., 2010)

S1.9 Primary CoQ_{10} deficiency due to mutations in the *COQ8B/ADCK4* gene (OMIM *615567) [# of patients: 88]

Gene [Patient ID]	# of patients (# of families)	Mutation	Level of CoQ_{10} (% of control)[1]	Age at onset (sex) if known	Symptoms	Biochemical tests and muscle pathology	RCC enzymes	CoQ_{10} dose and response[2]	Age at last reported exam or death	Corresponding PI	References
**COQ8B [1]**	2 (1)	R178W (HOM)	~ 11% (EBV-transformed lymphoblasts)	7 y/o	SRNS	no data	no data	no data	kidney transplant at age 10	Friedhelm Hildebrandt, Boston Children's Hospital, USA	(Ashraf et al., 2013)
**COQ8B [2]**		R178W (HOM)	~10% (EBV-transformed lymphoblasts)	13 y/o	SRNS	no data	no data	no data	kidney transplant at age 15		

**COQ8B [3]**	1 (1)	W34X/ T319dup (CH)	no data	10 y/o	SRNS	no data	no data	no data	kidney transplant at age 14		
**COQ8B [4]**	2 (1)	F215Lfs X14/R477Q (CH)	no data	13 y/o	SRNS	no data	no data	no data	kidney transplant at age 15		
**COQ8B [5]**		F215Lfs X14/R477Q (CH)	no data	12 y/o	SRNS	no data	no data	no data	kidney transplant at age 13		
**COQ8B [6]**	3 (1)	D286G/ E483X (CH)	no data	14 y/o	SRNS	no data	no data	no data	kidney transplant at age 18		
**COQ8B [7]**		D286G/ E483X (CH)	no data	3 y/o	SRNS	no data	no data	no data	3 years of age		
**COQ8B [8]**		D286G/ E483X (CH)	no data	9 y/o	SRNS	no data	no data	no data	9 years of age		
**COQ8B [9]**	2 (1)	R320W (HOM)	no data	12 y/o	SRNS	no data	no data	no data	ESRF at age 17		
**COQ8B [10]**		R320W (HOM)	no data	20 y/o	SRNS	no data	no data	no data	ESRF at age 23		
**COQ8B [11]**	2 (1)	R343W (HOM)	no data	20 y/o	SRNS	no data	no data	no data	ESRF at age 20		
**COQ8B [12]**		R343W (HOM)	no data	18 y/o	SRNS	no data	no data	no data	ESRF at age 19		
**COQ8B [13]**	2 (1)	Q452Hfs (HOM)	~ 27% (fibroblasts)	16 y/o	SRNS	no data	no data	no data	16 years of age		
**COQ8B [14]**		Q452Hfs (HOM)	~ 27% (fibroblasts)	21 y/o	SRNS	no data	no data	no data	21 years of age		
**COQ8B [15]**	1 (1)	H400Nfs X11 (HOM)	~ 8% (EBV-transformed lymphoblasts)	< 1 y/o	SRNS	no data	no data	no data	no data		
**COQ8B [16]**	1 (1)	R178W (HOM)	no data	30 y/o (F)	NS/FSGS	proteinuria, hypoalbuminemia, uPCR↑	no data	20 mg/kg/day, a decrease in uPCR and stabilization of eGFR *[Obj.]*	no data	Toshiki Doi, Hiroshima University Hospital, Japan	(Maeoka et al., 2020)
**COQ8B [17]**	5 (1)	E447Gfs X10 (HOM)	no data	14 y/o (M)	SRNS/FSGS	no data	no data	no data	ESRF at the age of 17.7	Beata S. Lipska-Zietkiewicz, Medical University of Gdansk, Poland	(Atmaca et al., 2017; Korkmaz et al., 2016)
**COQ8B [18]**		E447Gfs X10 (HOM)	no data	7.3 y/o (F)	SRNS/FSGS, epilepsy	no data	no data	no data	ESRF at the age of 12.6		
**COQ8B [19]**		E447Gfs X10 (HOM)	no data	17 y/o (F)	NS	no data	no data	no data	ESRF at the age of 18		

**COQ8B [20]**		E447Gfs X10 (HOM)	no data	27 y/o (F)	NS	no data	no data	no data	ESRF at the age of 31		
**COQ8B [21]**		E447Gfs X10 (HOM)	no data	7 y/o (F)	NS	no data	no data	no data	7 years of age		
**COQ8B [22]**	4 (1)	E447Gfs X10 (HOM)	no data	25.7 y/o (F)	SRNS/FSGS	no data	no data	20-30mg/kg/day for 3 months, response not described	37 years of age, ESRF at the age of 35.4		
**COQ8B [23]**		E447Gfs X10 (HOM)	no data	16.7 y/o (M)	NS	no data	no data	not treated	25.3 years of age, ESRF at the age of 16.7		
**COQ8B [24]**		E447Gfs X10 (HOM)	no data	13.5 y/o (M)	SRNS/FSGS	no data	no data	not treated	22.3 years of age, ESRF at the age of 16.6		
**COQ8B [25]**		not tested	no data	22 y/o (M)	NS	no data	no data	no data	ESRF at the age of 22		
**COQ8B [26]**	2 (1)	L98R (HOM)	no data	5.9 y/o (F)	NS/FSGS, primary nocturnal enuresis	no data	no data	no data	5.9 years of age		
**COQ8B [27]**		L98R (HOM)	no data	13.3 y/o (M)	NS/FSGS, primary nocturnal enuresis	no data	no data	no data	ESRF at the age of 14		
**COQ8B [28]**	2 (1)	R178W (HOM)	no data	14.3 y/o (M)	NS/FSGS, hypermetropia, astigmatism	no data	no data	no data	ESRF at the age of 14.3		
**COQ8B [29]**		R178W (HOM)	no data	9.8 y/o (M)	NS/FSGS, hypermetropia, astigmatism	no data	no data	no data	ESRF at the age of 9.8		
**COQ8B [30]**	2 (1)	L98R (HOM)	no data	13.5 y/o (F)	NS/FSGS, lupus-like symptoms	no data	no data	20-30mg/kg/day for 22 months, response not described	20.3 years of age		
**COQ8B [31]**		L98R (HOM)	no data	27 y/o (F)	NS/FSGS	no data	no data	20-30mg/kg/day, response not described	30 years of age		
**COQ8B [32]**	4 (1)	E447Gfs X10 (HOM)	no data	14.9 y/o (M)	NS	no data	no data	no data	ESRF at the age of 14.9		
**COQ8B [33]**		E447Gfs X10 (HOM)	no data	13.2 y/o (F)	NS, epilepsy	no data	no data	no data	ESRF at the age of 13.2		
**COQ8B [34]**		E447Gfs X10 (HOM)	no data	18 y/o (M)	NS	no data	no data	no data	ESRF at the age of 18		
**COQ8B [35]**		E447Gfs X10 (HOM)	no data	9 y/o (M)	NS	no data	no data	treated, dosage and response not described	9 years of age		
**COQ8B [36]**	2 (1)	D250N (HOM)	no data	16.9 y/o (M)	SRNS/FSGS	no data	no data	no data	ESRF at the age of 17.4		
**COQ8B [37]**		D250N (HOM)	no data	13.4 y/o (F)	SRNS/FSGS	no data	no data	no data	ESRF at the age of 13.7		

**COQ8B [38]**	1 (1)	F215Lfs X14 (HOM)	no data	15.1 y/o (M)	SRNS/FSGS	no data	no data	no data	ESRF at the age of 15.8		
**COQ8B [39]**	1 (1)	H400Nfs X11 (HOM)	no data	10.8 y/o (M)	SRNS/FSGS	no data	no data	no data	ESRF at the age of 15.9		
**COQ8B [40]**	1 (1)	P310L/ A498E (CH)	no data	5.1 y/o (F)	NS/FSGS, seizure	no data	no data	no data	ESRF at the age of 13.6		
**COQ8B [41]**	1 (1)	F215Lfs X14 (HOM)	no data	14.2 y/o (M)	NS/FSGS, retinitis pigmentosa, hypospadias	no data	no data	no data	ESRF at the age of 13.6		
**COQ8B [42]**	1 (1)	E447Gfs X10 (HOM)	no data	17.6 y/o (M)	SRNS/FSGS	no data	no data	no data	ESRF at the age of 18		
**COQ8B [43]**	1 (1)	E81X/ R490C CH)	no data	11 y/o (M)	steroid-resistant nephrotic-level proteinuria	no data	no data	no data	normal renal function at age of 12	Friedhelm Hildebrandt, Boston Children's Hospital, USA	(Wang et al., 2017a)
**COQ8B [44]**	1 (1)	R150X/ D250H (CH)	no data	8 y/o (F)	SRNS/FSGS	no data	no data	no data	ESRF at the age of 11.7		
**COQ8B [45]**	1 (1)	R178W/ D250H (CH)	no data	9 y/o (F)	SRNS	no data	no data	no data	ESRF at the age of 11		
**COQ8B [46]**	1 (1)	S246N (CH)	no data	8 y/o (F)	SRNS/FSGS	no data	no data	no data	normal renal function at age of 9		
**COQ8B [47]**	1 (1)	S246N (CH)	no data	17 y/o (F)	isolated proteinuria	no data	no data	no data	normal renal function at age of 18		
**COQ8B [48]**	1 (1)	D250H (HOM)	no data	10 days (F)	NS	no data	no data	no data	no data		
**COQ8B [49]**	1 (1)	D250H (HOM)	no data	1 y/o (F)	SRNS/FSGS	no data	no data	no data	ESRF at the age of 6		
**COQ8B [50]**	1 (1)	D250H/ Q365E (CH)	no data	6 y/o (F)	proteinuria/FSGS	no data	no data	no data	normal renal function at age of 12		
**COQ8B [51]**	2 (1)	P150Q/ N253K (CH)	no data	8 y/o (F)	SRNS/FSGS	no data	no data	not treated	ESRF at the age of 15	Hae Il Cheong, Seoul National University Children’s Hospital, South Korea	(Park et al., 2017b)
**COQ8B [52]**		P150Q/ N253K (CH)	no data	5 y/o (M)	SRNS	no data	no data	not treated	ESRF at the age of 10		
**COQ8B [53]**	1 (1)	S246N/ N253K (CH)	no data	10 y/o (F)	SRNS/FSGS	no data	no data	not treated	ESRF at the age of 13		
**COQ8B [54]**	1 (1)	S246N (HOM)	no data	10 y/o (F)	SRNS/FSGS	no data	no data	not treated	ESRF at the age of 12		

**COQ8B [55]**	1 (1)	S246N/ R490C (CH)	no data	6 y/o (F)	SRNS/FSGS	no data	no data	not treated	ESRF at the age of 10		
**COQ8B [56]**	1 (1)	S246N (HOM)	no data	12 y/o (F)	NS	no data	no data	△complete remission of proteinuria with cyclosporine treatment; after diagnosis of primary CoQ deficiency, started on CoQ_{10} (30mg/kg/day) with simultaneously tapering doses of steroid and cyclosporine, response not described	13.5 years of age		
**COQ8B [57]**	1 (1)	D209H/ C306X (CH)	no data	14 y/o (M)	NS/FSGS	no data	no data	150mg/day, a very limited reduction in the severity of urine protein/creatine ratio after 3 months of treatment *[NR]*	no data	Zhangxue Hu, West China Hospital, China	(Yang et al., 2018)
**COQ8B [58]**	1 (1)	D250Y/ A217T (CH)	no data	5 y/o (M)	SRNS/FSGS	no data	no data	no data	ESRF at the age of 10	Benedetta Chiodini, Université Libre de Bruxelles, Belgium	(Lolin et al., 2017)
**COQ8B [59]**	4 (1)	H400Qfs X11 (HOM)	no data	18 y/o (M)	non-nephrotic proteinuria, CKD	no data	no data	not treated	died at the age of 29	Fatih Ozaltin, Hacettepe University, Turkey	(Atmaca et al., 2017)
**COQ8B [60]**		H400Qfs X11 (HOM)	no data	12 y/o (M)	NS, CKD, seizure	no data	no data	20-30mg/kg/day for 13 months, response not described	13.5 years of age		
**COQ8B [61]**		H400Qfs X11 (HOM)	no data	2 y/o (F)	NS	no data	no data	20-30mg/kg/day for 10 months, a decrease of proteinuria but no change of eGFR	4.5 years of age		
**COQ8B [62]**		H400Qfs X11 (HOM)	no data	7 y/o (M)	non-nephrotic proteinuria	no data	no data	20-30mg/kg/day for 10 months, a decrease of proteinuria but no change of eGFR	8.5 years of age		
**COQ8B [63]**	2 (1)	H400Qfs X11 (HOM)	no data	13 y/o (F)	non-nephrotic proteinuria, CKD	no data	no data	20-30mg/kg/day for 17 months, response not described	22.4 years of age		
**COQ8B [64]**		H400Qfs X11 (HOM)	no data	5 y/o (M)	nephrotic syndrome, CKD	no data	no data	20-30mg/kg/day for 17 months, response not described	16.5 years of age		
**COQ8B [65]**	1 (1)	E447Gfs X11 (HOM)	no data	12 y/o (F)	NS	no data	no data	not treated	died at 14.8 years of age		
**COQ8B [66]**	5 (1)	H400Qfs X11 (HOM)	no data	17.7 y/o (F)	NS	no data	no data	not treated	died at 21.1 years of age		

**COQ8B [67]**		H400Qfs X11 (HOM)	no data	4.2 y/o (M)	non-nephrotic proteinuria	no data	no data	20-30mg/kg/day for 12 months, response not described	18.3 years of age		
**COQ8B [68]**		H400Qfs X11 (HOM)	no data	22.6 y/o (F)	NS, CKD	no data	no data	not treated	26 years of age		
**COQ8B [69]**		H400Qfs X11 (HOM)	no data	7.7 y/o (F)	NS	no data	no data	20-30mg/kg/day for 14 months, response not described	11 years of age		
**COQ8B [70]**		H400Qfs X11 (HOM)	no data	23.7 y/o (F)	non-nephrotic proteinuria	no data	no data	20-30mg/kg/day for 10 months, a decrease of proteinuria but no change of eGFR	24.6 years of age		
**COQ8B [71]**	3 (1)	E447Gfs X11 (HOM)	no data	12,4 y/o (F)	protéinurie, ESRF, cardiomyopathy	no data	no data	20-30mg/kg/day for 15 months, response not described	15.5 years of age		
**COQ8B [72]**		E447Gfs X11 (HOM)	no data	9.6 y/o (F)	NS, CKD	no data	no data	20-30mg/kg/day for 22 months, response not described	12.8 years of age		
**COQ8B [73]**		E447Gfs X11 (HOM)	no data	20.3 y/o (F)	non-nephrotic proteinuria, ESKD	no data	no data	20-30mg/kg/day for 10 months, response not described	25.3 years of age		
**COQ8B [74]**	1 (1)	R477Q (HOM)	no data	17.8 y/o (F)	CKD, autism, hypothy/ooidism, intellectual impairment	no data	no data	20-30mg/kg/day for 11 months, no response *[NR]*	20.3 years of age		
**COQ8B [75]**	1 (1)	K98R (HOM)	no data	9 y/o (F)	non-nephrotic proteinuria	no data	no data	20-30mg/kg/day for 21 months, a decrease of proteinuria but no change in eGFR	18.5 years of age		
**COQ8B [76]**	3 (1)	E447Gfs X10 (HOM)	no data	16.4 y/o (F)	NS, CKD	no data	no data	20-30mg/kg/day for 17 months, response not described	18 years of age		
**COQ8B [77]**		E447Gfs X10 (HOM)	no data	6.4 y/o (M)	CKD, seizure	no data	no data	20-30mg/kg/day for 16 months, response not described	26.5 years of age		
**COQ8B [78]**		E447Gfs X10 (HOM)	no data	24 y/o (M)	non-nephrotic proteinuria	no data	no data	20-30mg/kg/day for 13 months, a decrease of proteinuria but no change of eGFR	25.3 years of age		
**COQ8B [79]**	3 (1)	K98R (HOM)	no data	9 y/o (M)	NS	no data	no data	20-30mg/kg/day for 12 months, response not described	16.3 years of age		
**COQ8B [80]**		K98R (HOM)	no data	9.6 y/o (M)	non-nephrotic proteinuria, ESKF, pulmonary hypertension	no data	no data	20-30mg/kg/day for 12 months, response not described	11 years of age		
**COQ8B [81]**		K98R (HOM)	no data	32.2 y/o (M)	CKD, pulmonary hypertension	no data	no data	20-30mg/kg/day for 13 months, a decrease of proteinuria but an increase of eGFR	39 years of age		

**COQ8B [82]**	1 (1)	D250H/ R178W (CH)	no data	9 m/o (F)	proteinuria	no data	no data	15 to 30mg/kg/day, a reduction of urine protein at 1-year follow-up *[Obj.]*	no data	Jianhua Mao, The Second Hospital of Jiaxing, China	(Feng et al., 2017)
**COQ8B [83]**	1 (1)	D209H/ S205N (CH)	no data	11 y/o (F)	NS/FSGS, proteinuria	no data	no data	15 to 30mg/kg/day, no response (proteinuria was persistent, and serum creatine and urea nitrogen were increased at 1-year follow up) *[NR]*	no data		
**COQ8B [84]**	2 (1)	COQ8B (D250H, HOM) NPHS1 (E447K, HOM)	no data	9 y/o (F)	SRNS/FSGS, dyspnea, weakness, cardiac dysfunction	anemia, proteinuria, blood BUN and creatinine↑, hypo-albuminemia	no data	△Dosage is not described, given with metoprolol tartrate, losartan potassium, and peritoneal dialysis. At a 2-years follow-up, renal dysfunction was persistent but remained stable, while heart function showed no improvement.	11 years of age	Huijie Xiao, Peking University, China	(Zhang et al., 2017)
**COQ8B [85]**		COQ8B (D250H, HOM) NPHS1 (E447K, HOM)	no data	2 y/o (M)	SRNS/FSGS	proteinuria, blood BUN↑, hypo-albuminemia	no data	After the genetic diagnosis, prednisone and tacrolimus were withdrawn and CoQ_{10} treatment started. Renal function showed a slight increase at 2-years follow-up *[NR]*	2.6 years of age		
**COQ8B [86]**	1 (1)	R91C/ S246N (HOM)	no data	3 y/o (M)	Isolated (non-nephrotic) proteinuria	proteinuria, normal eGFR	no data	15 mg/kg/day, a decrease of proteinuria within 4-months follow-up *[Obj.]*	no data	Li Zhang, The First Hospital of Jilin University, China	(Zhai et al., 2020)
**COQ8B [87]**	1 (1)	I346S/ W520X (CH)	no data	5 y/o (F)	FSGS, proteinuria, rhabdomyolysis	uPCR↑	no data	2100/day since the age of 18 years, developed ESRD a year later *[NR]*	ESRD at 19 years of age	Asmaa S. AbuMaziad, University of Arizona, USA	(AbuMaziad et al., 2021)
**COQ8B [88]**	1 (1)	D250N (HOM)	no data	adolescence (F)	nephropathy, kidney failure	serum creatinine↑	no data	not treated	kidney transplant at the age of 23	Mohd Fareed, CSIR Indian Institute of Integrative Medicine, India	(Fareed et al., 2021)

S.1.10 Primary CoQ_{10} deficiency-5 (COQ10D5; 614654) due to mutations in the *COQ9* gene [# of patients: 3]

Gene [Patient ID]	# of patients (# of families)	Mutation	Level of CoQ_{10} (% of control)[1]	Age at onset (sex) if known	Symptoms	Biochemical tests and muscle pathology	RCC enzymes	CoQ_{10} dose and responses[2]	Age at last reported exam or death	Corresponding PI	References
**COQ9 [1]**	1 (1)	R244X (HOM)	~ 15% (muscle) ~ 18% (fibroblasts)	neonatal (M)	renal tubulopathy, ventricular hypertrophy, seizure, cerebellar atrophy, development delay	blood lactate level↑, type IIB fiber atrophy and lipid accumulation in the muscle	CII+CIII↓ (muscle)	initiated at 11.5 months of age at the dose of 60mg/day and increasing to 300mg/day (after 6 days) which was continued until the patient's death, no response *[NR]*	died at the age of 2 years	Shamima Rahman, Great Ormond Street Hospital, UK	(Duncan et al., 2009; Quinzii and Hirano, 2010; Quinzii et al., 2010; Rahman et al., 2001)
**COQ9 [2]**	1 (1)	S127_R202del (HOM)	~ 11% (fibroblasts)	neonatal (M)	hypotonia, bradycardia, encephalopathy	blood lactate level↑, blood alanine↑	CII+CIII↓ (skin)	not treated	died at 18 days of life	H Prokisch, TUM, Germany	(Danhauser et al., 2016)
**COQ9 [3]**	1 (1)	G129VfsX17 (HOM)	no data	4 m/o (F)	seizure, hypotonia, dysmorphic features, growth retardation, microcephaly	no data	no data	initiated at 10 months of age at the dose of 5mg/kg/day and increasing to 50mg/kg/day after the genetic diagnosis, no response *[NR]*	9 months of age	Asburce Olgac, University of Health Sciences, Turkey	(Olgac et al., 2020)

y/o: years old; m/o: months old; HOM: homozygous; HET: heterozygous; CH: compound heterozygous; CSF: cerebrospinal fluid; RCC: respiratory chain complex; CI: complex I; CII: complex II; CIII: complex III; CS: citrate synthase; COX: cytochrome c oxidase; CKD: chronic kidney disease; ICARS: The International Cooperative Ataxia Rating Scale; ETC: electron transport chain; NS: nephrotic syndrome; FSGS: focal segmental glomerulosclerosis; eGFR: estimated Glomerular Filtration Rate; ESRF: end-stage renal failure; ERG: electroretinography; SDH: succinate dehydrogenase; SRNS: steroid-resistant nephrotic syndrome; SND: sensorineural deafness; SARA: Scale for the Assessment and Rating of Ataxia; uPCR: urine protein creation ratio; del: deletion; fs: frameshift; dup: duplication; ins: insertion; delins: deletion-insertion.

[1] CoQ levels are shown as reported or as a percentage relative to the mean value of reported normal range; *[Obj.]*: counted as patients with an objective description of the response to CoQ_{10} treatment in **Table 2**, that is where quantitative or semi-quantitative measures were used to describe CoQ_{10} treatment effects; *[Subj.]*: counted as patients with a subjective description of the response to CoQ_{10} treatment in **Table 2**; *[NR]*: counted as non-responders in **Table 2**; △ reported being given simultaneously with other modifications.

Table S2 Cases excluded from the final analysis and reasons for their exclusion.

Gene [Patient ID*]	Mutation	Level of CoQ_{10} (% of control)[1]	Age at onset (sex) if known	Symptoms	CoQ_{10} dose and responses	Reason for exclusion	Reference
**COQ2 [1]**	R197H/ N228S (CH)	~ 36% (fibroblasts) <3% (kidney, muscle)	18 m/o (M)	SRNS	30mg/kg/day since age 21 months, response not described	lack of information	(Diomedi-Camassei et al., 2007; Quinzii et al., 2010)
**COQ2 [19]**	G390A (HOM)	no data	18 y/o (F)	SRNS/FSGS	treated, response not described	lack of information	(Gigante et al., 2017)
**COQ2 [20]**	G390A (HOM)	no data	16 y/o (F)	SRNS/FSGS	treated, response not described	lack of information	
**COQ4 [6]**	P64S (HOM)	~ 63% (muscle)	10 m/o (M)	motor deterioration, ataxia, epileptic seizures, swallowing impairment, progressive scoliosis, cognitive deterioration	treated, response not described	lack of information	(Brea-Calvo et al., 2015)
**COQ4 [31]**	G124S (HOM)	low (fibroblasts)	2 m/o (M)	encephalopathy, spasms, seizure, development delay	beginning at 7 years of age, dose not described, response not described	lack of information	(Yu et al., 2019)
**COQ6 [7]**	G255R (HOM)	no data	0.2 y/o	SRNS, SND, bilateral nephrolithiasis	30mg/kg/day beginning at 2 months of age (together with enalapril), a decrease of proteinuria, SND and severe growth retardation were noted at 10 months of age	co-treatment with other medication, thus impossible to judge CoQ_{10} treatment effectiveness	(Heeringa et al., 2011)
**COQ6 [18]**	P261L (HOM)	no data	0.8 y/o (M)	SRNS	treated, response not described	lack of information	(Gigante et al., 2017)
**COQ6 [25]**	A353D (HOM)	no data	5 y/o (M)	SRNS, SND, optic atrophy	15mg/kg/day of idebenone beginning at age of 17 years after the onset of optical symptoms, an improvement in the visual acuity after 2 months of treatment. After 13 months of treatment, the optical examination was stable, but the patient did not recover normal vision, still exhibiting persistent optic atrophy. After 3 years of treatment, minimal optic atrophy was reported. No change of the deafness status since treatment initiation. [other medications: immunosuppressive treatment]	insufficient information for judging treatment efficiency	(Justine Perrin et al., 2020)
**COQ6 [26]**	A353D (HOM)	no data	4 y/o (M)	SRNS, SND	10mg/kg/day of idebenone since age 7, after 13 months of treatment, hearing loss was not changed and renal involvement remained stable with only Enalapril, demonstrated by negative proteinuria.	insufficient information for judging treatment efficiency	(Justine Perrin et al., 2020)
**COQ8A [10]**	Y514C/ T584del (CH)	~ 51% (fibroblasts), ~ 46% (muscle)	5 y/o (M)	cerebellar ataxia, gynecomastia, feet and thumbs in dystonic position	60 -700 mg/day over 8 years, the patient reported mild subjective improvement, and stabilization of the cerebellar ataxia was observed on examination	insufficient information for judging treatment efficiency	(Lagier-Tourenne et al., 2008; Lamperti et al., 2003; Quinzii et al., 2010)

**COQ8A [17]**	R348X (HOM)	<14.5% (muscle)	6 y/o (F)	seizure, ataxia, cerebellar atrophy, a mild cognitive delay	10mg/kg/day initiated at the age of 8 years, within 6 months improvement of ataxia was observed, but after 5 years of treatment, MRI showed increased cerebellar atrophy	insufficient information for judging treatment efficiency	(Terracciano et al., 2012)
**COQ8A [19]**	T584delACC/ P502R (CH)	no data	childhood (F)	mild dysfluent speech and clumsiness, cerebellar atrophy, mild dysarthria	treated, dosage and response not described	lack of information	(Blumkin et al., 2014)
**COQ8A [26]**	S616LfsX114 (HOM)	no data	14 y/o (M)	cerebellar ataxia, myoclonus, tremors, dysarthric speech	200mg/day, improvement in speech and fatigue after 3 months of treatment	insufficient information for judging treatment efficiency	(Liu et al., 2014)
**COQ8A [41]**	A338V (HOM)	no data	13 y/o (F)	cerebellar ataxia, muscle weakness, myoclonus, tremor, dysarthria	dosage not described, improved tremors	insufficient information for judging treatment efficiency	(Traschutz et al., 2020)
**COQ8A [42]**	V83fs (HOM)	no data	8 y/o (F)	cerebellar ataxia, tremors	1250mg/day, improved tremors	insufficient information for judging treatment efficiency	(Traschutz et al., 2020)
**COQ8A [43]**	V83fs (HOM)	no data	16 y/o (M)	cerebellar ataxia, dysarthria, tremors	1250mg/day, response not described	lack of information	(Traschutz et al., 2020)
**COQ8A [44]**	T584del/ A338T (CH)	~ 15% (muscle)	6 y/o (F)	ataxia, pan-cerebellar atrophy	100mg/day, response not described	lack of information	(Traschutz et al., 2020)
**COQ8A [45]**	E481X (HOM)	no data	1 y/o (F)	ataxia, motor retardation, cognitive impairment, tremors	200mg/day, no initial apparent effect but after stop: fatigue and falls; improvement of muscle weakness with reintroduction of CoQ_{10}	insufficient information for judging treatment efficiency	(Traschutz et al., 2020)
**COQ8A [48]**	c.589-3C>G/ G615D (CH)	no data	2 y/o (F)	ataxia, hypotonia	10 mg/kg/day, improvement in stability	insufficient information for judging treatment efficiency	(Traschutz et al., 2020)
**COQ8A [50]**	c.589-3C>G/ R301W (CH)	no data	2 y/o (F)	ataxia, seizure	10 mg/kg/day, improved balance	insufficient information for judging treatment efficiency	(Traschutz et al., 2020)
**COQ8A [55]**	E568X (HOM)	no data	6 y/o (F)	spastic hypertonia, ataxia	300mg/day since 5 years old, more energetic, mentally quicker	insufficient information for judging treatment efficiency	(Traschutz et al., 2020)
**COQ8A [60]**	H85AfsX42 (HOM)	no data	3 y/o (M)	ataxia	400 - 1200 mg/day, response not described	lack of information	(Traschutz et al., 2020)
**COQ8A [74]**	G615D (HOM)	no data	childhood (M)	ataxia, dysmetria, seizure	135mg/day of idebenone for 9 months, response not described	lack of information	(Mignot et al., 2013)
**COQ8A [75]**	G615D (HOM)	no data	7 y/o (F)	ataxia. dysmetria	135mg/day of idebenone, response not described	lack of information	(Mignot et al., 2013)

**COQ8A [91]**	27.6 kb deletion of 1q42.3 involving exons 1 and 2 (HOM)	~ 34% (muscle), normal range (fibroblasts)	13 y/o (F)	ataxia, tremors, hand bradykinesia, subtle and variable speech dysfluency	Tremor improved on trihexyphenidyl/clonazepam combination therapy before ubiquinol supplementation which was initiated at age 19 years. Ubiquinol dosage was not described. After two years of ubiquinol and high-dose vitamin B-complex treatments, tremor was stable, and the patient was able to tandem walk normally. She had marked bradykinesia though.	insufficient information for judging treatment efficiency	(Galosi et al., 2019)
**COQ8A [92]**	G615D/ L197VfsX 20 (CH)	no data	7 y/o (F)	tremors, ataxia, dysmetria, difficulty writing and hand clumsiness	800mg/day initiated at the age of 8.5, clinical stabilization was reported after the treatment	insufficient information for judging treatment efficiency	(Galosi et al., 2019)
**COQ8A [94]**	R301W/ E446AfsX 33 (CH)	no data	3 y/o (M)	ataxia, speech difficulties, seizure, tremors, dystonia	10 mg/kg/day, initiated at age 10, but has been taken only intermittently, response not described	lack of information	(Galosi et al., 2019)
**COQ8A [97]**	c.655+1G >A/ A339T (CH)	no data	3 y/o (F)	exercise intolerance, dysarthria, seizure, stroke-like episodes, ataxia, homonymous hemianopsia, dysarthria	400mg/day, response not described	lack of information	https://doi.org/10.26815/acn.2020.00276
**COQ8A [105]**	A339T (HOM)	no data	14 m/o (F)	hypotonia, developmental delay, ataxia, glaucoma, dysmorphic features	100mg/day, response not described	lack of information	(Cotta et al., 2020)
**COQ8B [22]**	E447GfsX 10 (HOM)	no data	25.7 y/o (F)	SRNS/FSGS	20-30mg/kg/day for 3 months, response not described	lack of information	(Atmaca et al., 2017; Korkmaz et al., 2016)
**COQ8B [30]**	L98R (HOM)	no data	13.5 y/o (F)	NS/FSGS, lupus-like symptoms	20-30mg/kg/day for 22 months, response not described	lack of information	(Atmaca et al., 2017; Korkmaz et al., 2016)
**COQ8B [31]**	L98R (HOM)	no data	27 y/o (F)	NS/FSGS	20-30mg/kg/day, response not described	lack of information	(Atmaca et al., 2017; Korkmaz et al., 2016)
**COQ8B [35]**	E447GfsX 10 (HOM)	no data	9 y/o (M)	NS	treated, dosage and response not described	lack of information	(Atmaca et al., 2017; Korkmaz et al., 2016)
**COQ8B [56]**	S246N (HOM)	no data	12 y/o (F)	NS	complete remission of proteinuria with cyclosporine treatment; after diagnosis of primary CoQ deficiency, started on CoQ_{10} (30mg/kg/day) with simultaneously tapering doses of steroid and cyclosporine, response not described	lack of information	(Park et al., 2017b)
**COQ8B [60]**	H400QfsX 11 (HOM)	no data	12 y/o (M)	NS, CKD, seizure	20-30mg/kg/day for 13 months, response not described	lack of information	(Atmaca et al., 2017)
**COQ8B [61]**	H400QfsX 11 (HOM)	no data	2 y/o (F)	NS	20-30mg/kg/day for 10 months, a decrease of proteinuria but no change of eGFR		(Atmaca et al., 2017)

**COQ8B [62]**	H400QfsX 11 (HOM)	no data	7 y/o (M)	non-nephrotic proteinuria	20-30mg/kg/day for 10 months, a decrease of proteinuria but no change of eGFR		(Atmaca et al., 2017)
**COQ8B [63]**	H400QfsX 11 (HOM)	no data	13 y/o (F)	non-nephrotic proteinuria, CKD	20-30mg/kg/day for 17 months, response not described	lack of information	(Atmaca et al., 2017)
**COQ8B [64]**	H400QfsX 11 (HOM)	no data	5 y/o (M)	nephrotic syndrome, CKD	20-30mg/kg/day for 17 months, response not described	lack of information	(Atmaca et al., 2017)
**COQ8B [67]**	H400QfsX 11 (HOM)	no data	4.2 y/o (M)	non-nephrotic proteinuria	20-30mg/kg/day for 12 months, response not described	lack of information	(Atmaca et al., 2017)
**COQ8B [69]**	H400QfsX 11 (HOM)	no data	7.7 y/o (F)	NS	20-30mg/kg/day for 14 months, response not described	lack of information	(Atmaca et al., 2017)
**COQ8B [70]**	H400QfsX 11 (HOM)	no data	23.7 y/o (F)	non-nephrotic proteinuria	20-30mg/kg/day for 10 months, a decrease of proteinuria but no change of eGFR		(Atmaca et al., 2017)
**COQ8B [71]**	E447GfsX 11 (HOM)	no data	12,4 y/o (F)	proteinuria, ESRF, cardiomyopathy	20-30mg/kg/day for 15 months, response not described	lack of information	(Atmaca et al., 2017)
**COQ8B [72]**	E447GfsX 11 (HOM)	no data	9.6 y/o (F)	NS, CKD	20-30mg/kg/day for 22 months, response not described	lack of information	(Atmaca et al., 2017)
**COQ8B [73]**	E447GfsX 11 (HOM)	no data	20.3 y/o (F)	non-nephrotic proteinuria, ESKD	20-30mg/kg/day for 10 months, response not described	lack of information	(Atmaca et al., 2017)
**COQ8B [75]**	K98R (HOM)	no data	9 y/o (F)	non-nephrotic proteinuria	20-30mg/kg/day for 21 months, a decrease of proteinuria but no change in eGFR		(Atmaca et al., 2017)
**COQ8B [76]**	E447GfsX 10 (HOM)	no data	16.4 y/o (F)	NS, CKD	20-30mg/kg/day for 17 months, response not described	lack of information	(Atmaca et al., 2017)
**COQ8B [77]**	E447GfsX 10 (HOM)	no data	6.4 y/o (M)	CKD, seizure	20-30mg/kg/day for 16 months, response not described	lack of information	(Atmaca et al., 2017)
**COQ8B [78]**	E447GfsX 10 (HOM)	no data	24 y/o (M)	non-nephrotic proteinuria	20-30mg/kg/day for 13 months, a decrease of proteinuria but no change of eGFR		(Atmaca et al., 2017)
**COQ8B [79]**	K98R (HOM)	no data	9 y/o (M)	NS	20-30mg/kg/day for 12 months, response not described	lack of information	(Atmaca et al., 2017)
**COQ8B [80]**	K98R (HOM)	no data	9.6 y/o (M)	non-nephrotic proteinuria, ESKF, pulmonary hypertension	20-30mg/kg/day for 12 months, response not described	lack of information	(Atmaca et al., 2017)
**COQ8B [81]**	K98R (HOM)	no data	32.2 y/o (M)	CKD, pulmonary hypertension	20-30mg/kg/day for 13 months, a decrease of proteinuria but an increase of eGFR	not possible to judge treatment efficiency	(Atmaca et al., 2017)
**COQ8B [84]**	COQ8B (D250H, HOM) NPHS1	no data	9 y/o (F)	SRNS/FSGS, dyspnea, weakness, cardiac dysfunction	dosage is not described, given with metoprolol tartrate, losartan potassium, and peritoneal dialysis. At a 2-years follow-up, renal dysfunction was	insufficient information for judging treatment efficiency	(Zhang et al., 2017)

	(E447K, HOM)				persistent but remained stable, while heart function showed no improvement.		

[*] Patient IDs are the same as in **Table S1**. [1] CoQ levels are shown as reported or as a percentage relative to the mean value of reported normal range; y/o: years old; m/o: months old; HOM: homozygous; HET: heterozygous; CH: compound heterozygous; CKD: chronic kidney disease; ICARS: The International Cooperative Ataxia Rating Scale; NS: nephrotic syndrome; FSGS: focal segmental glomerulosclerosis; eGFR: estimated Glomerular Filtration Rate; ESRF: end-stage renal failure; SRNS: steroid-resistant nephrotic syndrome; SND: sensorineural deafness; SARA: Scale for the Assessment and Rating of Ataxia; uPCR: urine protein creation ratio; del: deletion; fs: frameshift; dup: duplication; ins: insertion; delins: deletion-insertion.

Table S3 Partial effects reported for CoQ10 treatment of primary CoQ deficiency patients.

Gene	Total No. of treated patients [a]	Effects not described or uncertain [a]	No. of patients included in the analysis	Responding		Not responding
				Objective description	Subjective description	
PDSS1	0	-	0	-	-	-
PDSS2	2	0	2	0	0	2
COQ2	10	3	7	1	0	6
COQ4	21	2	19	3	2	14
COQ5	3	0	3	3	0	0
COQ6	5[2]	2[2]	3	1	0	2
COQ7	3	0	3	0	0	3
COQ8A/ADCK3	59[2]	18[2]	41	9	2	30
COQ8B/ADCK4	33	24	9	3	0	6
COQ9	2	0	2	0	0	2

Treatment effects established by quantitative or semi-quantitative measures to describe the response to CoQ_{10} treatment were counted as responding with objective description, while descriptions of convincingly positive effects but without relying on a quantitative or semi-quantitative measure were counted as responding with subjective description. "Not responding" include the patients who were reported not to respond to CoQ_{10} treatment or whose responses we consider lacking a convincing demonstration of a response to CoQ_{10} supplementation. [a] The number of patients treated with the CoQ derivative idebenone are indicated as superscripts.

Table S4 Patient cases classified as not responding to CoQ_{10} treatment.

Gene [Patient ID[*]]	Mutation	Level of CoQ_{10} (% of control[1])	Age at onset (sex) if known	Symptoms	CoQ_{10} dose and responses	Note	Reference
**PDSS2 [1]**	Q332X/ S382L (CH)	~ 14% (muscle) ~ 12% (fibroblasts)	3 m/o (M)	NS, hypotonia, Leigh syndrome, seizure	50mg/day beginning at age 3 months, no response, died at age of 8 months *[NR]*	infantile patient with multisystem illnesses, no response observed	(Lopez et al., 2006; Quinzii et al., 2008; Salviati et al., 2012)
**PDSS2 [4]**	H162R/ c.1042_1148-2816del (CH)	no data	neonatal (M)	NS, encephalomyopathy, hypertrophic cardiomyopathy, deafness, retinitis pigmentosa, global developmental delay	20mg/kg/day, no response, died at age of 8 months (1 month after admission) *[NR]*	infantile patient with multisystem illnesses, no response observed	(Ivanyi et al., 2018)

**COQ2 [3]**	Y297C (HOM)	~18% (fibroblasts) ~ 37.5% (muscle)	11 m/o (M)	infantile encephalomyopathy, SRNS/FSGS, hypotonia, optic atrophy, tremors, psychomotor regression	30 mg/kg/day beginning at age 22months, neurologic picture improved, but no change in renal function *[NR]*	minimal and ambiguous effects	(Diomedi-Camassei et al., 2007; Montini et al., 2008; Quinzii et al., 2006; Quinzii et al., 2008; Salviati et al., 2005)
**COQ2 [4]**	Y297C (HOM)	~ 17% (fibroblasts)	12 m/o (F)	NS/FSGS without any clinical signs of neurologic involvement.	30 mg/kg/day, there was no improvement during the first 2 weeks of treatment; an episode of acute renal failure required continuous hemofiltration for 4 days. 20 days after the initiation of the treatment, recovery of renal function and a reduced level of proteinuria was observed. After 50 months of therapy, renal function remains normal, though proteinuria was still present (other medication: diuretics) *[NR]*	minimal and ambiguous effects	(Diomedi-Camassei et al., 2007; Montini et al., 2008; Quinzii et al., 2006; Quinzii et al., 2008; Salviati et al., 2005)
**COQ2 [9]**	S109N (HOM)	~ 11.4% (fibroblasts)	neonatal (M)	peripheral hypertonia, cardiomyopathy, hypertrophic cardiomegaly, nephrotic syndrome	30mg/kg/day, no response, died at age of 5 months *[NR]*	infantile patient with multisystem illnesses, no response	(Scalais et al., 2013; Ziosi et al., 2017)
**COQ2 [21]**	c.288dupC/ R126G (CH)	no data	25 y/o (M)	diffuse glomerulosclerosis, end-stage nephropathy, retinopathy	30 mg/kg/day for 6 months, no ERG improvement, but best corrected visual acuity and areas of retinal atrophy on autofluorescence were noted to be stable on treatment *[NR]*	a minimal and ambiguous effect	(Abdelhakim et al., 2020)
**COQ2 [22]**	c.288dupC/ R126G (CH)	no data	21 y/o (M)	mesangial sclerosis, end-stage nephropathy, retinopathy, lymphoma			
**COQ2 [23]**	c.288dupC/ R126G (CH)	no data	23 y/o (F)	retinopathy, end-stage nephropathy			
**COQ4 [7]**	L82Q/ R158Q (CH)	~ 16% (muscle)	neonatal (F)	seizures, severe lactic and respiratory acidosis, heart failure	20 mg/kg/day beginning at the first day of life, which resulted in normalization of lactate and improvement in cardiac function. Nevertheless, the patient continued exhibiting intermittent episodes of lactic acidemia and cardiac decompensation until death (other medications: thiamine, riboflavin, hydroxocobalamin, biotin), died at 2 months of age *[NR]*	infantile patient, died shortly despite several treatment attempts	(Chung et al., 2015)
**COQ4 [12]**	R240C (HOM)	no data	neonatal (F)	poor/absent reflexes, cardiac hypertrophy, left hip dysplasia, hypotonia, episodes of apnea and bradycardia	15 mg/kg/day beginning at age 1 month, no response [other medications: pyridoxal phosphate, folinic acid, and riboflavin], died at 7 weeks old *[NR]*	infantile patient, died shortly	
**COQ4 [14]**	T77I (HOM)	no data	4 y/o (M)	tremors, dysarthria, seizure, spastic tetraparesis and ataxia	1000mg/day beginning at age 13, the 6 min walk test was stable over the period of a year *[NR]*	a minimal and ambiguous effect	(Bosch et al., 2018)
**COQ4 [15]**	T77I (HOM)	~ 22% (fibroblasts)	9 y/o (F)	seizure, dysarthria, spastic tetraparesis, ataxia	1000mg/day beginning at age 11, the 6 min walk test was stable over a year, developed a second stroke-like episode at age 14 *[NR]*	a minimal and ambiguous effect	

**COQ4 [17]**	G124S (HOM)	~ 50% (fibroblasts)	neonatal (F)	motor deterioration, weak responsiveness, dystonia, nystagmus, respiratory distress, seizure	50 mg/kg/day starting at the age of 12 months, improvement in seizure, screaming, and respiratory distress, no improvement in nystagmus, dystonia, psychomotor development, and ambulation *[NR]*	minimal and ambiguous effects	(Lu et al., 2019)
**COQ4 [19]**	G95D/ R102H (CH)	~ 98% (fibroblasts)	5 y/o (F)	cognitive impairment, dysmetria, spastic ataxia, seizure	100mg/kg/day of ubiquinol, no response after 6 months (as assessed by the SARA scale) *[NR]*	no response observed	(Mero et al., 2021)
**COQ4 [23]**	G124S/ c.402+1G>C (CH)	low (fibroblasts)	neonatal (M)	encephalopathy, cardiomyopathy, visual and hearing impairment, respiratory failure, apnea, developmental delay	40 mg/kg/day beginning at 5 months of age, poor response, died at 8 months of age *[NR]*	infantile patient with multisystem illness, died shortly	(Yu et al., 2019)
**COQ4 [24]**	G124S/ c.402+1G>C (CH)	no data	neonatal (M)	cardiomyopathy, respiratory distress, metabolic acidosis	15 mg/kg/day, no response [other medication: carnitine], died at 2.5 days of age *[NR]*	infantile patient, died shortly after birth	
**COQ4 [25]**	G124S (HOM)	no data	neonatal (F)	cardiomyopathy, seizure, developmental delay	treated, dose not described, cardiac function improved gradually and normalized after 10 days [other medication: intravenous immunoglobulin] *[NR]*	a minimal effect	
**COQ4 [26]**	G124S/ c.402+1G>C (CH)	no data	neonatal (F)	seizure, apnea, encephalopathy, cardiomyopathy	started at the age of 4 years and 5 months, dose not described, no response observed after 1 month of treatment *[NR]*	no response observed after 1 month of treatment	
**COQ4 [27]**		no data	2 m/o (F)	seizure, respiratory distress, cardiomegaly	started at 1 year of age, dose not described, no response, passed away 1 month later *[NR]*	infantile patient, died shortly after start of CoQ_{10} treatment	
**COQ4 [28]**	W184R/ c.402+1G>C (CH)	low (fibroblasts)	8 m/o (M)	microcephaly, developmental delay, dystonia, visual impairment, oro-motor dysfunction	dose not described, no response *[NR]*	no response observed	
**COQ4 [28]**	G124S (HOM)	low (fibroblasts)	infancy (F)	visual impairment, dystonia, spasticity, developmental delay	since 2 years old, dose not described, no response, died at 3.5 years of age *[NR]*	died while on CoQ_{10} treatment	
**COQ4 [30]**	G124V/ G124S (CH)	low (fibroblasts)	infancy (F)	encephalopathy, dystonia, spasticity, developmental delay, visual impairment, seizure	beginning at 9 months of age, dose not described, subjective improvement in response [other medication: levetiracetam] *[NR]*	a minimal effect	
**COQ6 [6]**	G255R (HOM)	no data	0.3 y/o	SRNS, SND, facial dysmorphism	100mg/day, improvement of SND *[NR]*	a minimal effect	(Heeringa et al., 2011)
**COQ6 [17]**	R360W/ c.804delC (CH)	no data	2 y/o (F)	steroid-resistant glomerulopathy, poor growth	30 mg/kg/day, remission of glomerulopathy after 1 month of treatment, growth acceleration after 12 months and a reduction of respiratory airway infections *[NR]*	minimal and ambiguous effects	(Koyun et al., 2019; Stanczyk et al., 2018)
**COQ7 [1]**	V141E (HOM)	~ 10% (fibroblasts, muscle)	neonatal (M)	muscular hypotonia, developmental retardation, learning disabilities, hearing impairment, visual dysfunction, not able to sit and walk independently	initially treated with idebenone, switched to COQ_{10} after the diagnosis of a primary CoQ_{10} deficiency (around age of 10 years), dosage unknown, stalling the regression and significantly reducing the pain were noted *[NR]*	minimal effects	(Freyer et al., 2015)
**COQ7 [2]**	L111P (HOM)	~ 70% (fibroblasts)	14 m/o (F)	spasticity, muscle wasting, inability to walk without support	22.8 mg/kg/day, no response after 3 months of treatment *[NR]*	no response observed	(Wang et al., 2017b)

**COQ7 [3]**	K200IfsX56/ R107W (CH)	~ 12% (fibroblasts)	neonatal (M)	cardiomyopathy, growth retardation, hypotonia, ptosis, visual impairment, hearing impairment, muscle weakness, infantile spasms	beginning at 2 months of age, and the dose was increased to 20 mg/kg/day at 12 months of life, the patient died around the same time *[NR]*	infantile patient with multisystem illness, no response	(Kwong et al, 2019)
**COQ8A [1]**	R213W/ G272V (CH)	~ 29% (muscle)	18 m/o (F)	hypotonia, *talus valgus*, developmental delay, seizure, ataxia, epilepsia partialis continua	20 mg/kg/day (350mg/day) for 8 years, no response *[NR]*	no response observed	(Mignot et al., 2013; Mollet et al., 2008)
**COQ8A [2]**	R213W/ G272V (CH)	no data	2 y/o (F)	hypotonia, seizure, ataxia, developmental delay	350mg/day for 13 months, no response *[NR]*	no response observed	
**COQ8A [3]**	E551K (HOM)	~ 8% (muscle), normal range (fibroblasts)	18 m/o (M)	cerebella ataxia, strabismus, muscle weakness, trunk hypotonia, tonic seizure	5mg/kg/day from age 3 years, 10mg/kg/day from age 4 to 7, no response; followed by 10mg/kg/day of idebenone for 7 months which worsened the patient's conditions *[NR]*	no response observed	(Mollet et al., 2008)
**COQ8A [21]**	R271C/ A304T (CH)	normal range (muscle)	15 y/o (F)	cerebellar ataxia, tremors	300 mg/day, no response after 6 months *[NR]*	no response observed	(Horvath et al., 2012)
**COQ8A [22]**	A304V (HOM)	~ 8% (muscle)	27 y/o (F)	cerebellar ataxia, upper-limb myoclonus, seizure, dysmetria, cataract	300 mg/day, no response after 6 months *[NR]*	no response observed	
**COQ8A [23]**	R299W (HOM)	no data	1 y/o (F)	cerebellar ataxia, seizure, mental retardation, unable to walk by 12 years	200 mg/day, no response within 2 months *[NR]*	no response observed	
**COQ8A [24]**	Y429C/?	~ 22% (muscle)	1.5-2 y/o (F)	ataxia, muscle weakness, cognitive impairment, horizontal nystagmus, bilateral dysmetria, tremors	200 mg/day, no response within 2 months *[NR]*	no response observed	
**COQ8A [29]**	D305Y (HOM)	low (muscle)	5 y/o (M)	developmental delay, intellectual disability, ataxia, isolated pan-cerebellar features including head titubation, dysmetria, dysdiadochokinesia	800mg/day, inconsistent use for 2 years, no response *[NR]*	no response observed	
**COQ8A [38]**	A339T/ Y361 (CH)	no data	42 y/o (M)	cerebellar ataxia, stroke-like episode, muscle weakness, hearing loss	dosage not described, no response *[NR]*	no response observed	
**COQ8A [39]**	A337T (HOM)	no data	6 y/o (M)	cerebellar ataxia, dystonia, tremor,	600mg/day, no response *[NR]*	no response observed	
**COQ8A [51]**	R301W/ E446AfsX33 (CH)	low (muscle)	3 y/o (M)	ataxia	10 mg/kg/day, no response *[NR]*	no response observed	
**COQ8A [52]**	R301W/ E446AfsTer33 (CH)	no data	2 y/o (M)	ataxia, developmental retardation	10 mg/kg/day, no response *[NR]*	no response observed	
**COQ8A [53]**	R348X (HOM)	low (muscle)	10 y/o (F)	epilepsy, ataxia	600mg/day, no response *[NR]*	no response observed	
**COQ8A [54]**	R301W (HOM)	low (muscle)	8 y/o (F)	ataxia, seizure, cardiomyopathy	400mg/day, no response *[NR]*	no response observed	
**COQ8A [78]**	R299W/ R410X (CH)	no data	4 y/o (F)	ataxia, dysmetria, seizure	300mg/day for1 month, withdrawn, reversible side effect of treatment (anorexia) *[NR]*	no response observed	

**COQ8A [79]**	R299W/ R410X (CH)	no data	4 y/o (M)	ataxia, dysmetria, seizure	300mg/day for1 month, withdrawn, reversible side effect of treatment (diarrhea) *[NR]*	no response observed	
**COQ8A [80]**	R271C (HOM)	low (plasma)	1.5 y/o (F)	ataxia, seizure, dystonia, chorea, dysmetria, myoclonus, spasticity	30 mg/kg/day for 3 years, no response *[NR]*	no response observed	
**COQ8A [81]**	L197VfsX20 (HOM)	no data	19 y/o (F)	ataxia. dysmetria	1200mg/day for 1 year no response *[NR]*	no response observed	
**COQ8A [82]**	L197VfsX20 (HOM)	no data	19 y/o (F)	ataxia. dysmetria, seizure	1200mg/day for 1 year, no response *[NR]*	no response observed	
**COQ8A [83]**	Q360_Y361ins X (HOM)	no data	2 y/o (F)	ataxia. Dysmetria, tremors	800mg/day for 1 year, no response *[NR]*	no response observed	
**COQ8A [84]**	R299W (HOM)	~ 10-24% (muscle)	7 y/o (F)	ataxia, seizure, tremor	900mg/day for 6 months, no response *[NR]*	no response observed	(Hikmat et al., 2016)
**COQ8A [87]**	R299W (HOM)	no data	2 y/o (F)	ataxia, epilepsy, seizure, feeding difficulties	1000mg/day of deoxyubiquinone (probably ubiquinol) since age of 18, no response *[NR]*	no response observed	
**COQ8A [96]**	L277P/ c.1506+1G>A (CH)	normal range (plasma)	childhood (F)	ataxia	20 mg/kg/day, minimal improvement in an ataxia assessment score at 1-year follow-up *[NR]*	minimal effects	(Jacobsen et al., 2018)
**COQ8A [103]**	c.656-1G>T (HOM)	no data	20 y/o (F)	ataxia, writer's cramp	60mg/day of ubiquinol, initiated at 20 years old, stopped after only 2 months due to incompliance, no response *[NR]*	no response observed	(Amprosi et al., 2021)
**COQ8A [104]**	c.656-1G>T (HOM)	no data	7 y/o (M)	ataxia, writer's cramp	60mg/day of ubiquinol, initiated at 25 years old, due to adverse event (frequent headache); switched to 5mg/kg/day of CoQ_{10}; no response at 1-year follow-up *[NR]*	no response observed	
**COQ8A [107]**	R301W/ E446AfsX33 (CH)	no data	3 y/o (M)	ataxia, tremors, epilepsy, mild intellectual retardation	15 mg/kg/day for 6 months, no improvement in motor performance (Timed 25-foot walk test, SARA)	no response observed	(Schirinzi et al., 2019)
**COQ8A [108]**	R301W/ E446AfsX33 (CH)	no data	3 y/o (M)	ataxia, mild intellectual retardation	15 mg/kg/day for 6 months, no improvement in motor performance (Timed 25-foot walk test, SARA)	no response observed	
**COQ8A [109]**	G615D/ L197VfsX20 (CH)	no data	6 y/o (F)	ataxia, tremors	15 mg/kg/day for 1 year, improvement in Timed 25-foot walk but no significant change in SARA, gait analysis parameters and 6 min walking test	minimal and ambiguous effects	
**COQ8A [110]**	R301W/ c.589-3C > G (splice) (CH)	no data	2 y/o (F)	epilepsy, mild intellectual retardation	15 mg/kg/day for 1 year, improvement in Timed 25-foot walk but no significant change in SARA, gait analysis parameters and 6 min walking test	minimal and ambiguous effects	
**COQ8A [111]**	G27C (HOM)	no data	2 y/o (F)	seizure, developmental regression, hypothyroidism, mitral regurgitation, mitral valve prolapse, cerebellar atrophy, and epilepsia partialis continua	treated with CoQ_{10} after 11 years of age, dosage unknown, no effect on seizure frequency *[NR]*	no response observed	(Ashrafi et al., 2022)
**COQ8B [57]**	D209H/ C306X (CH)	no data	14 y/o (M)	NS/FSGS	150mg/day, a very limited reduction in the severity of urine protein/creatine ratio after 3 months of treatment *[NR]*	a minimal effect	(Yang et al., 2018)

**COQ8B [74]**	R477Q (HOM)	no data	17.8 y/o (F)	CKD, autism, hypothyroidism, intellectual impairment	20-30mg/kg/day for 11 months, no response *[NR]*	no response observed	(Atmaca et al., 2017)
**COQ8B [83]**	D209H/ S205N (CH)	no data	11 y/o (F)	NS/FSGS, proteinuria	15 to 30mg/kg/day, proteinuria was persistent, and serum creatine and urea nitrogen were increased at 1-year follow up *[NR]*	no response observed	(Feng et al., 2017)
**COQ8B [84]**	COQ8B (D250H, HOM) NPHS1 (E447K, HOM)	no data	9 y/o (F)	SRNS/FSGS, dyspnea, weakness, cardiac dysfunction	dosage is not described, given with metoprolol tartrate, losartan potassium, and peritoneal dialysis. At a 2-years follow-up, renal dysfunction was persistent but remained stable, while heart function showed no improvement *[NR]*	no response observed	(Zhang et al., 2017)
**COQ8B [85]**	COQ8B (D250H, HOM) NPHS1 (E447K, HOM)	no data	2 y/o (M)	SRNS/FSGS	After the genetic diagnosis, prednisone and tacrolimus were withdrawn and CoQ_{10} treatment started. Renal function showed a slight increase at 2-years follow-up *[NR]*	a minimal effect	
**COQ8B [87]**	I346S/ W520X (CH)	no data	5 y/o (F)	FSGS, proteinuria, rhabdomyolysis	2100mg/day since the age of 18 years, developed ESRD a year later *[NR]*	no effect on disease progression	(AbuMaziad et al., 2021)
**COQ9 [1]**	R244X (HOM)	~ 15% (muscle) ~ 18% (fibroblasts)	neonatal (M)	renal tubulopathy, ventricular hypertrophy, seizure, cerebellar atrophy, development delay	initiated at 11.5 months of age at the dose of 60mg/day and increasing to 300mg/day (after 6 days) which was continued until the patient's death, no response *[NR]*	no response observed	(Duncan et al., 2009; Quinzii and Hirano, 2010; Quinzii et al., 2010; Rahman et al., 2001)
**COQ9 [3]**	G129VfsX17 (HOM)	no data	4 m/o (F)	seizure, hypotonia, dysmorphic features, growth retardation, microcephaly	initiated at 10 months of age at the dose of 5mg/kg/day and increasing to 50mg/kg/day after the genetic diagnosis, no response *[NR]*	no response observed	(Olgac et al., 2020)

[*] Patient IDs are the same as in **Table S1**. [1] CoQ levels are shown as reported or as a percentage relative to the mean value of reported normal range; y/o: years old; m/o: months old; HOM: homozygous; HET: heterozygous; CH: compound heterozygous; CSF: cerebrospinal fluid; RCC: respiratory chain complex; CI: complex I; CII: complex II; CIII: complex III; CS: citrate synthase; COX: cytochrome c oxidase; CKD: chronic kidney disease; ICARS: The International Cooperative Ataxia Rating Scale; ETC: electron transport chain; NS: nephrotic syndrome; FSGS: focal segmental glomerulosclerosis; eGFR: estimated Glomerular Filtration Rate; ESRF: end-stage renal failure; ERG: electroretinography; SDH: succinate dehydrogenase; SRNS: steroid-resistant nephrotic syndrome; SND: sensorineural deafness; SARA: Scale for the Assessment and Rating of Ataxia; uPCR: urine protein creation ratio; del: deletion; fs: frameshift; dup: duplication; ins: insertion; delins: deletion-insertion.

Table S5 Cases with positive outcomes following CoQ_{10} treatment, classified as responding.

Gene [Patient ID*]	Mutation	Level of CoQ_{10} (% of control)[1]	Age at onset (sex) if known	Symptoms	CoQ_{10} dose and responses	Category of description of CoQ_{10} treatment effects	Reference
**COQ2 [25]**	Y353C/ T325A (CH)	no data	7 m/o (F)	SRNS	30 mg/kg/ day beginning at age 11 months, urinary protein decreased with the increasing dose of CoQ_{10}, and an increase of serum albumin, now on the dosage of 600mg/day *[Obj.]*	objective, as a decrease of proteinuria was reported	(Li et al., 2021)
**COQ4 [1]**	mono-allelic deletion (CH)	~ 43% (fibroblasts)	neonatal (M)	dysmorphic features, mental retardation, encephalomyopathy	30 mg/kg/day, improvement in physical status and social function. Conditions worsened (weakness and diffuse myalgia) after formulation change and dosage reduction to 2mg/kg/day. Remission of symptoms within a week after reverting back to the original dosage. Then switched to 15mg/kg/day of ubiquinol *[Obj.]*	objective, loss of response after treatment interruption and remission after resuming CoQ_{10} treatment	(Salviati et al., 2012)
**COQ4 [18]**	P193S/ R240C (CH)	~ 95% (fibroblasts)	2.5 y/o (M)	developmental delay, hypotonia, sialorrhea, spasticity, ataxia	30 mg/kg/day of ubiquinol, improvement in neuromuscular symptoms after 2 months, further improvement of motor skills in the following months, but speech delay and cognitive impairment persisted *[Subj.]*	subjective, as improvement of more than one symptom was reported	(Mero et al., 2021)
**COQ4 [20]**	G55V (CH)	normal range (blood)	8 y/o (M)	ataxia, spasticity, epilepsy, cognitive deterioration, dysarthria, dysmetria and dysdiadochokinesia	2000 mg/day, improvement of SARA score, dysarthria is persistent *[obj.]*	objective, as improvement of SARA score was reported	(Caglayan et al., 2019)
**COQ4 [21]**		normal range (blood)	8 y/o (F)	dysarthria, spastic ataxia, epilepsy, cognitive deterioration, dysmetria, dysdiadochokinesia	Treated, dose not described, improvement of SARA score, gait difficulty and dysarthria are persistent *[obj.]*	objective, as improvement of SARA score was reported	
**COQ4 [32]**	G124S (HOM)	no data	2 m/o (F)	hypotonia, developmental delay, bilateral cortical blinding, seizure, cardiomyopathy	30mg/kg/day beginning at 11 months of age, some improvement in seizure control and development *[Subj.]*	subjective, as improvement of more than one symptom was reported	
**COQ5 [1]**	biallelic duplication of last 4 exons	~ 57% (muscle) ~ 50% (leukocytes)	childhood (F)	ataxia, dysarthria, seizures, cognitive disability, behavioral problems, epilepsy, myoclonus, dysarthric cerebellar speech, dysmetria, mild tremors and mild lower limb spasticity	dose not described, improvement of ICARS scoring after 3 months, the patient appeared to have a quicker response rate during conversation and better alertness *[Obj.]*	objective, as improvement of SARA score was reported	(Malicdan et al., 2018)
**COQ5 [2]**		~ 66% (leukocytes)	childhood (F)	mild static gait ataxia, mild dysarthria, mild dysmetria and oculomotor apraxia, and horizontal nystagmus	dose not described, improvement of ICARS scoring after 3 months, the patient appeared to have a quicker response rate during conversation and better alertness *[Obj.]*	objective, as improvement of ICAR score was reported	
**COQ5 [3]**		~ 60% (leukocytes)	childhood (F)	mild motor delay, mild learning difficulties, mild cerebellar ataxia, mild cerebellar	dose not described, improvement of ICARS scoring after 3 months, the patient appeared to have a quicker response rate during conversation and better alertness *[Obj.]*	objective, as improvement of ICAR score was reported	

				dysarthria and horizontal nystagmus			
**COQ6 [9]**	A353D (HOM)	no data	2.5 y/o	SRNS, SND	beginning at age 5.5 years, dose not described, decrease of proteinuria but no hearing improvement, reoccurrence of proteinuria after temporary cessation of CoQ_{10} treatment and it decreased again after the treatment resumed *[Obj.]*	objective, loss of response after treatment interruption and remission after resuming CoQ_{10} treatment	
**COQ8A [4]**	G272D/ Q605GfsX125 (CH)	< 5% (muscle), normal range (fibroblasts)	3 y/o (F)	exercise intolerance, muscle weakness, cerebellar syndromes, seizure	6 mg/kg/day (750mg/day) of CoQ_{10} and L-carnitine were initiated at age 5, improved exercise tolerance and fewer vomiting episodes were noted after 3 months of therapy. CoQ_{10} was replaced with idebenone (5mg/kg/day) at the age of 9 years, and within the following 4 months, severe exercise intolerance reappeared with numerous episodes of vomiting. Reverting to CoQ_{10} treatment resulted in returns to the previous clinical status within 3 months. *[Obj.]*	objective, loss of response after treatment interruption and remission after resuming CoQ_{10} treatment	(Aure et al., 2004; Mignot et al., 2013; Mollet et al., 2008)
**COQ8A [18]**	T584delACC/ P502R (CH)	no data	2 y/o (F)	cerebellar ataxia, dysarthria, nystagmus, cognitive decline, psychiatric disorder	20mg/kg/day initiated at age 5, partial improvement in motor skills, balance, and strength; after 6 years, treatment was discontinued, and the patient's condition deteriorated. *[Obj.]*	objective, as improvement in more than symptom was reported and the patient's condition worsened after stopping CoQ_{10} treatment	(Blumkin et al., 2014)
**COQ8A [20]**	S616LfsX114/ R301Q (CH)	~ 45% (plasma)	9 y/o (M)	exercise intolerance, cerebellar ataxia, tremors, dysautonomia	120mg/day, self-reported fatigue and exercise tolerance improved after 2 weeks of therapy. After 2 years of therapy, ataxia and head tremor diminished and SARA total score improved. When the treatment was stopped for a month, the patient's condition deteriorated, rendering him to resume taking CoQ_{10}. *[Obj.]*	objective, as improvement of more than one symptom, including SARA score, was reported; and the patient' condition worsened after stopping CoQ_{10} treatment	(Zhang et al., 2020)
**COQ8A [25]**	S616LfsX114 (HOM)	~ 35% (fibroblasts)	10 y/o (F)	cerebellar ataxia, myoclonus, slurred speech, wheelchair-dependent by 30 years of age	400mg/day, improvement in myoclonic symptoms, speech quality (after 3 months), and ataxia with a reduction in SARA (after 6 months) *[Obj.]*	objective, as improvement of more than one symptom, including SARA score, was reported	(Liu et al., 2014)
**COQ8A [27]**	R301W/ c.1399-3_ 1408del (CH)	low (muscle)	11 y/o (M)	reduced dexterity, dysarthria, hypometric saccades, scanning speech, and dystonic posturing, tremors, ataxia	800mg/day, a resolution of tremors and improvement of limb and truncal dystonia after 9 months of treatment *[Subj.]*	subjective, as improvement of more than one symptom was reported	(Chang et al., 2018)
**COQ8A [28]**	T584del/ T511M (CH)	low (muscle)	10 y/o (F)	ataxia, tremors, dysarthria, appendicular dysmetria, truncal instability, titubation,	800mg/day, improvement of ataxia overall with a reduction in SARA score, able to work independently, after 9 months of therapy. *[Obj.]*	objective, as improvement of more than one symptom,	

				wheelchair-dependent by 53 years of age		including SARA score, was reported	
**COQ8A [76]**	del exons 3-15/ F508S (CH)	no data	6 y/o (M)	ataxia. dysmetria, myoclonus	300mg/day for 15 months, improvement in movement disorder and SARA score *[Obj.]*	objective, as improvement of more than one symptom, including SARA score, was reported	
**COQ8A [77]**	R299W/ L453RfsX24 (CH)	normal range (fibroblasts)	15 y/o (M)	ataxia, seizure, myoclonus, dysmetria	300mg/day for 8 months, improvement in movement disorder *[Subj.]*	subjective, as improvement of more than one symptom was reported	
**COQ8A [85]**	R299W/ F578V (CH)	~ 34-60% (muscle)	7 y/o (M)	ataxia, seizure, dysmetria, tremors, dysarthria, dysdiadochokinesia	600mg/day since the age of 33, improvement in balance and coordination (reported by the patient) and a reduction of SARA score *[Obj.]*	objective, as improvement of more than one symptom, including SARA score, was reported	
**COQ8A [95]**	L277P/ c.1506+1G>A (CH)	low (muscle) normal range (plasma)	childhood (F)	ataxia, dysmetria, hypotonia	20 mg/kg/day, improvement in an ataxia assessment score at 1-year follow-up *[Obj.]*	objective, as improvement of an ataxia assessment score was reported	(Jacobsen et al., 2018)
**COQ8A [112]**	L609V (HET)	moderate deficiency in fibroblasts and muscle	unknown (F)	ataxia	30mg/kg/day from 8 years old, a reduction in ICARS after years of treatment *[Obj.]*	objective, as improvement of an ataxia assessment score was reported	(Pineda et al., 2010)
**COQ8B [16]**	R178W (HOM)	no data	30 y/o (F)	NS/FSGS	20 mg/kg/day, a decrease in uPCR and stabilization of eGFR *[Obj.]*	objective, as improvement of more than one symptom, including uPCR was reported	(Maeoka et al., 2020)
**COQ8B [82]**	D250H/ R178W (CH)	no data	9 m/o (F)	proteinuria	15 to 30mg/kg/day, a reduction of urine protein at 1-year follow-up *[Obj.]*	objective, as improvement of proteinuria was reported	(Feng et al., 2017)
**COQ8B [86]**	R91C/ S246N (HOM)	no data	3 y/o (M)	Isolated (non-nephrotic) proteinuria	15 mg/kg/day, a decrease of proteinuria within 4-months follow-up *[Obj.]*	objective, as improvement of proteinuria was reported	(Zhai et al., 2020)

[*] Patient IDs are the same as in **Table S1**. [1] CoQ levels are shown as reported or as a percentage relative to the mean value of reported normal range; y/o: years old; m/o: months old; HOM: homozygous; HET: heterozygous; CH: compound heterozygous; ICARS: The International Cooperative Ataxia Rating Scale; NS: nephrotic syndrome; FSGS: focal segmental glomerulosclerosis; eGFR: estimated Glomerular Filtration Rate; SRNS: steroid-resistant nephrotic syndrome; SND: sensorineural deafness; SARA: Scale for the Assessment and Rating of Ataxia; uPCR: urine protein creation ratio; del: deletion; fs: frameshift; dup: duplication; ins: insertion; delins: deletion-insertion.

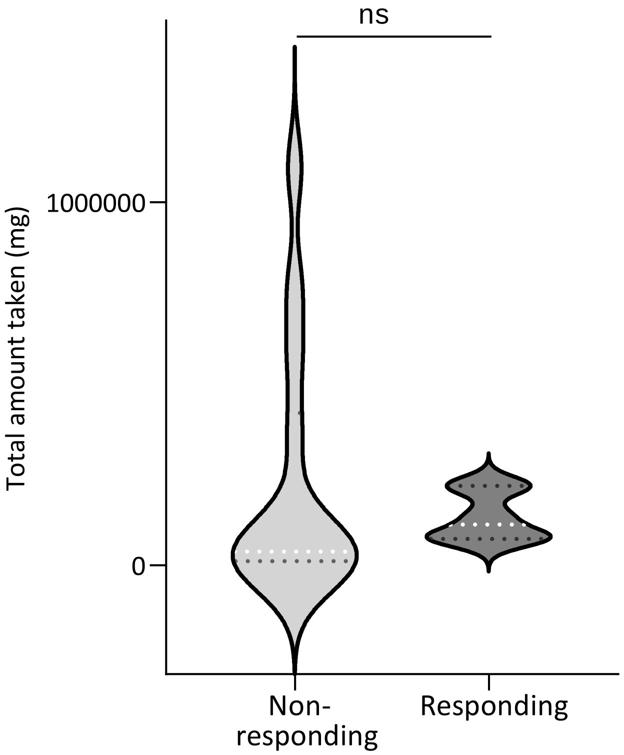

Fig. S1 The violin plot of total CoQ_{10} amounts taken. The amounts were calculated as dosage/day x duration. Only the treatments for which CoQ_{10} dosages were reported as mg/day and durations were also reported are included in this analysis (14 patients in the non-responding group and 6 patients in the responding group). ns: not significant (Student's *t*-test). Of note, because of the scale of the Y-axis, the median of total CoQ_{10} taken for the non-responding group looks small and close to zero.

References

Abdelhakim, A.H., A.V. Dharmadhikari, S.D. Ragi, J.R.L. de Carvalho, Jr., C.L. Xu, A.L. Thomas, C.M. Buchovecky, M.M. Mansukhani, A.B. Naini, J. Liao, V. Jobanputra, I.H. Maumenee, and S.H. Tsang. 2020. Compound heterozygous inheritance of two novel COQ2 variants results in familial coenzyme Q deficiency. *Orphanet J Rare Dis*. 15:320.

AbuMaziad, A.S., T.M. Thaker, T.M. Tomasiak, C.C. Chong, M.K. Galindo, and H.E. Hoyme. 2021. The role of novel COQ8B mutations in glomerulopathy and related kidney defects. *American journal of medical genetics. Part A*. 185:60-67.

Amprosi, M., M. Zech, R. Steiger, W. Nachbauer, A. Eigentler, E.R. Gizewski, M. Guger, E. Indelicato, and S. Boesch. 2021. Familial writer's cramp: a clinical clue for inherited coenzyme Q10 deficiency. *Neurogenetics*. 22:81-86.

Ashraf, S., H.Y. Gee, S. Woerner, L.X. Xie, V. Vega-Warner, S. Lovric, H. Fang, X. Song, D.C. Cattran, C. Avila-Casado, A.D. Paterson, P. Nitschke, C. Bole-Feysot, P. Cochat, J. Esteve-Rudd, B. Haberberger, S.J. Allen, W. Zhou, R. Airik, E.A. Otto, M. Barua, M.H. Al-Hamed, J.A. Kari, J. Evans, A. Bierzynska, M.A. Saleem, D. Bockenhauer, R. Kleta, S. El Desoky, D.O. Hacihamdioglu, F. Gok, J. Washburn, R.C. Wiggins, M. Choi, R.P. Lifton, S. Levy, Z. Han, L. Salviati, H. Prokisch, D.S. Williams, M. Pollak, C.F. Clarke, Y. Pei, C. Antignac, and F. Hildebrandt. 2013. ADCK4 mutations promote steroid-resistant nephrotic syndrome through CoQ10 biosynthesis disruption. *J Clin Invest*. 123:5179-5189.

Ashrafi, M.R., R. Haghighi, R.S. Badv, H. Ghabeli, A.R. Tavasoli, E. Pourbakhtyaran, Z. Rezaei, N. Mahdieh, P. Mohammadi, and M. Heidari. 2022. Epilepsia Partialis Continua a Clinical Feature of a Missense Variant in the ADCK3 Gene and Poor Response to Therapy. *J Mol Neurosci*.

Atmaca, M., B. Gulhan, E. Korkmaz, M. Inozu, O. Soylemezoglu, C. Candan, A.K. Bayazit, A.M. Elmaci, G. Parmaksiz, A. Duzova, N. Besbas, R. Topaloglu, and F. Ozaltin. 2017. Follow-up results of patients with ADCK4 mutations and the efficacy of CoQ10 treatment. *Pediatr Nephrol*. 32:1369-1375.

Aure, K., J.F. Benoist, H. Ogier de Baulny, N.B. Romero, O. Rigal, and A. Lombes. 2004. Progression despite replacement of a myopathic form of coenzyme Q10 defect. *Neurology*. 63:727-729.

Blumkin, L., E. Leshinsky-Silver, A. Zerem, K. Yosovich, T. Lerman-Sagie, and D. Lev. 2014. Heterozygous Mutations in the ADCK3 Gene in Siblings with Cerebellar Atrophy and Extreme Phenotypic Variability. *JIMD Rep*. 12:103-107.

Bosch, A.M., E.J. Kamsteeg, R.J. Rodenburg, A.W. van Deutekom, D.R. Buis, M. Engelen, and J.M. Cobben. 2018. Coenzyme Q10 deficiency due to a COQ4 gene defect causes childhood-onset spinocerebellar ataxia and stroke-like episodes. *Mol Genet Metab Rep*. 17:19-21.

Brea-Calvo, G., T.B. Haack, D. Karall, A. Ohtake, F. Invernizzi, R. Carrozzo, L. Kremer, S. Dusi, C. Fauth, S. Scholl-Burgi, E. Graf, U. Ahting, N. Resta, N. Laforgia, D. Verrigni, Y. Okazaki, M. Kohda, D. Martinelli, P. Freisinger, T.M. Strom, T. Meitinger, C. Lamperti, A. Lacson, P. Navas, J.A. Mayr, E. Bertini, K. Murayama, M. Zeviani, H. Prokisch, and D. Ghezzi. 2015. COQ4

mutations cause a broad spectrum of mitochondrial disorders associated with CoQ10 deficiency. *Am J Hum Genet*. 96:309-317.
Bujan, N., A. Arias, R. Montero, J. Garcia-Villoria, W. Lissens, S. Seneca, C. Espinos, P. Navas, L. De Meirleir, R. Artuch, P. Briones, and A. Ribes. 2014. Characterization of CoQ10 biosynthesis in fibroblasts of patients with primary and secondary CoQ10 deficiency. *Journal of inherited metabolic disease*. 37:53-62.
Caglayan, A.O., H. Gumus, E. Sandford, T.L. Kubisiak, Q. Ma, A.B. Ozel, H. Per, J.Z. Li, V.G. Shakkottai, and M. Burmeister. 2019. COQ4 Mutation Leads to Childhood-Onset Ataxia Improved by CoQ10 Administration. *Cerebellum*. 18:665-669.
Chang, A., M. Ruiz-Lopez, E. Slow, M. Tarnopolsky, A.E. Lang, and R.P. Munhoz. 2018. ADCK3-related Coenzyme Q10 Deficiency: A Potentially Treatable Genetic Disease. *Mov Disord Clin Pract*. 5:635-639.
Cheng, H.L., Y.R. Shao, Y. Dong, H.L. Dong, L. Yang, Y. Ma, Y. Shen, and Z.Y. Wu. 2021. Genetic spectrum and clinical features in a cohort of Chinese patients with autosomal recessive cerebellar ataxias. *Transl Neurodegener*. 10:40.
Chung, W.K., K. Martin, C. Jalas, S.R. Braddock, J. Juusola, K.G. Monaghan, B. Warner, S. Franks, M. Yudkoff, L. Lulis, R.H. Rhodes, V. Prasad, E. Torti, M.T. Cho, and M. Shinawi. 2015. Mutations in COQ4, an essential component of coenzyme Q biosynthesis, cause lethal neonatal mitochondrial encephalomyopathy. *J Med Genet*. 52:627-635.
Cotta, A., C.L. Alston, S. Baptista-Junior, J.F. Paim, E. Carvalho, M.M. Navarro, M. Appleton, Y.S. Ng, J. Valicek, A.L. da-Cunha-Junior, M.I. Lima, A. de la Rocque Ferreira, R.I. Takata, I.P. Hargreaves, G.S. Gorman, R. McFarland, G. Pierre, and R.W. Taylor. 2020. Early-onset coenzyme Q10 deficiency associated with ataxia and respiratory chain dysfunction due to novel pathogenic COQ8A variants, including a large intragenic deletion. *JIMD Rep*. 54:45-53.
Danhauser, K., D. Herebian, T.B. Haack, R.J. Rodenburg, T.M. Strom, T. Meitinger, D. Klee, E. Mayatepek, H. Prokisch, and F. Distelmaier. 2016. Fatal neonatal encephalopathy and lactic acidosis caused by a homozygous loss-of-function variant in COQ9. *Eur J Hum Genet*. 24:450-454.
Dinwiddie, D.L., L.D. Smith, N.A. Miller, A.M. Atherton, E.G. Farrow, M.E. Strenk, S.E. Soden, C.J. Saunders, and S.F. Kingsmore. 2013. Diagnosis of mitochondrial disorders by concomitant next-generation sequencing of the exome and mitochondrial genome. *Genomics*. 102:148-156.
Diomedi-Camassei, F., S. Di Giandomenico, F.M. Santorelli, G. Caridi, F. Piemonte, G. Montini, G.M. Ghiggeri, L. Murer, L. Barisoni, A. Pastore, A.O. Muda, M.L. Valente, E. Bertini, and F. Emma. 2007. COQ2 nephropathy: a newly described inherited mitochondriopathy with primary renal involvement. *J Am Soc Nephrol*. 18:2773-2780.
Duncan, A.J., M. Bitner-Glindzicz, B. Meunier, H. Costello, I.P. Hargreaves, L.C. Lopez, M. Hirano, C.M. Quinzii, M.I. Sadowski, J. Hardy, A. Singleton, P.T. Clayton, and S. Rahman. 2009. A nonsense mutation in COQ9 causes autosomal-recessive neonatal-onset primary coenzyme Q10 deficiency: a potentially treatable form of mitochondrial disease. *Am J Hum Genet*. 84:558-566.
Fareed, M., V. Makkar, R. Angral, M. Afzal, and G. Singh. 2021. Whole-exome sequencing reveals a novel homozygous mutation in the COQ8B gene associated with nephrotic syndrome. *Scientific reports*. 11:13337.

Feng, C., Q. Wang, J. Wang, F. Liu, H. Shen, H. Fu, and J. Mao. 2017. Coenzyme Q10 supplementation therapy for 2 children with proteinuria renal disease and ADCK4 mutation: Case reports and literature review. *Medicine (Baltimore)*. 96:e8880.

Freyer, C., H. Stranneheim, K. Naess, A. Mourier, A. Felser, C. Maffezzini, N. Lesko, H. Bruhn, M. Engvall, R. Wibom, M. Barbaro, Y. Hinze, M. Magnusson, R. Andeer, R.H. Zetterstrom, U. von Dobeln, A. Wredenberg, and A. Wedell. 2015. Rescue of primary ubiquinone deficiency due to a novel COQ7 defect using 2,4-dihydroxybensoic acid. *J Med Genet*.

Galosi, S., E. Barca, R. Carrozzo, T. Schirinzi, C.M. Quinzii, M. Lieto, G. Vasco, G. Zanni, M. Di Nottia, D. Galatolo, A. Filla, E. Bertini, F.M. Santorelli, V. Leuzzi, R. Haas, M. Hirano, and J. Friedman. 2019. Dystonia-Ataxia with early handwriting deterioration in COQ8A mutation carriers: A case series and literature review. *Parkinsonism Relat Disord*. 68:8-16.

Gerards, M., B. van den Bosch, C. Calis, K. Schoonderwoerd, K. van Engelen, M. Tijssen, R. de Coo, A. van der Kooi, and H. Smeets. 2010. Nonsense mutations in CABC1/ADCK3 cause progressive cerebellar ataxia and atrophy. *Mitochondrion*. 10:510-515.

Gigante, M., S. Diella, L. Santangelo, E. Trevisson, M.J. Acosta, M. Amatruda, G. Finzi, G. Caridi, L. Murer, M. Accetturo, E. Ranieri, G.M. Ghiggeri, M. Giordano, G. Grandaliano, L. Salviati, and L. Gesualdo. 2017. Further phenotypic heterogeneity of CoQ10 deficiency associated with steroid resistant nephrotic syndrome and novel COQ2 and COQ6 variants. *Clin Genet*. 92:224-226.

Heeringa, S.F., G. Chernin, M. Chaki, W. Zhou, A.J. Sloan, Z. Ji, L.X. Xie, L. Salviati, T.W. Hurd, V. Vega-Warner, P.D. Killen, Y. Raphael, S. Ashraf, B. Ovunc, D.S. Schoeb, H.M. McLaughlin, R. Airik, C.N. Vlangos, R. Gbadegesin, B. Hinkes, P. Saisawat, E. Trevisson, M. Doimo, A. Casarin, V. Pertegato, G. Giorgi, H. Prokisch, A. Rotig, G. Nurnberg, C. Becker, S. Wang, F. Ozaltin, R. Topaloglu, A. Bakkaloglu, S.A. Bakkaloglu, D. Muller, A. Beissert, S. Mir, A. Berdeli, S. Varpizen, M. Zenker, V. Matejas, C. Santos-Ocana, P. Navas, T. Kusakabe, A. Kispert, S. Akman, N.A. Soliman, S. Krick, P. Mundel, J. Reiser, P. Nurnberg, C.F. Clarke, R.C. Wiggins, C. Faul, and F. Hildebrandt. 2011. COQ6 mutations in human patients produce nephrotic syndrome with sensorineural deafness. *J Clin Invest*. 121:2013-2024.

Hikmat, O., C. Tzoulis, P.M. Knappskog, S. Johansson, H. Boman, P. Sztromwasser, E. Lien, E. Brodtkorb, D. Ghezzi, and L.A. Bindoff. 2016. ADCK3 mutations with epilepsy, stroke-like episodes and ataxia: a POLG mimic? *Eur J Neurol*. 23:1188-1194.

Horvath, R., B. Czermin, S. Gulati, S. Demuth, G. Houge, A. Pyle, C. Dineiger, E.L. Blakely, A. Hassani, C. Foley, M. Brodhun, K. Storm, J. Kirschner, G.S. Gorman, H. Lochmuller, E. Holinski-Feder, R.W. Taylor, and P.F. Chinnery. 2012. Adult-onset cerebellar ataxia due to mutations in CABC1/ADCK3. *J Neurol Neurosurg Psychiatry*. 83:174-178.

Ivanyi, B., G.Z. Racz, P. Gal, K. Brinyiczki, I. Bodi, T. Kalmar, Z. Maroti, and C. Bereczki. 2018. Diffuse mesangial sclerosis in a PDSS2 mutation-induced coenzyme Q10 deficiency. *Pediatr Nephrol*. 33:439-446.

Jacobsen, J.C., W. Whitford, B. Swan, J. Taylor, D.R. Love, R. Hill, S. Molyneux, P.M. George, R. Mackay, S.P. Robertson, R.G. Snell, and K. Lehnert. 2018. Compound Heterozygous Inheritance of Mutations in Coenzyme Q8A Results in Autosomal Recessive Cerebellar Ataxia and Coenzyme Q10 Deficiency in a Female Sib-Pair. *JIMD Rep*. 42:31-36.

Jakobs, B.S., L.P. van den Heuvel, R.J. Smeets, M.C. de Vries, S. Hien, T. Schaible, J.A. Smeitink, R.A. Wevers, S.B. Wortmann, and R.J. Rodenburg. 2013. A novel mutation in COQ2 leading to fatal infantile multisystem disease. *Journal of the neurological sciences*. 326:24-28.

Justine Perrin, R., C. Rousset-Rouviere, F. Garaix, A. Cano, J. Conrath, O. Boyer, and M. Tsimaratos. 2020. COQ6 mutation in patients with nephrotic syndrome, sensorineural deafness, and optic atrophy. *JIMD Rep*. 54:37-44.

Korkmaz, E., B.S. Lipska-Zietkiewicz, O. Boyer, O. Gribouval, C. Fourrage, M. Tabatabaei, S. Schnaidt, S. Gucer, F. Kaymaz, M. Arici, A. Dinckan, S. Mir, A.K. Bayazit, S. Emre, A. Balat, L. Rees, R. Shroff, C. Bergmann, C. Mourani, C. Antignac, F. Ozaltin, F. Schaefer, and C. PodoNet. 2016. ADCK4-Associated Glomerulopathy Causes Adolescence-Onset FSGS. *J Am Soc Nephrol*. 27:63-68.

Koyun, M., E. Comak, and S. Akman. 2019. CoenzymeQ10 therapy in two sisters with CoQ6 mutations with long-term follow-up. *Pediatr Nephrol*. 34:737-738.

Lagier-Tourenne, C., M. Tazir, L.C. Lopez, C.M. Quinzii, M. Assoum, N. Drouot, C. Busso, S. Makri, L. Ali-Pacha, T. Benhassine, M. Anheim, D.R. Lynch, C. Thibault, F. Plewniak, L. Bianchetti, C. Tranchant, O. Poch, S. DiMauro, J.L. Mandel, M.H. Barros, M. Hirano, and M. Koenig. 2008. ADCK3, an ancestral kinase, is mutated in a form of recessive ataxia associated with coenzyme Q10 deficiency. *Am J Hum Genet*. 82:661-672.

Lamperti, C., A. Naini, M. Hirano, D.C. De Vivo, E. Bertini, S. Servidei, M. Valeriani, D. Lynch, B. Banwell, M. Berg, T. Dubrovsky, C. Chiriboga, C. Angelini, E. Pegoraro, and S. DiMauro. 2003. Cerebellar ataxia and coenzyme Q10 deficiency. *Neurology*. 60:1206-1208.

Li, M., Z. Yue, H. Lin, H. Wang, H. Chen, and L. Sun. 2021. COQ2 mutation associated isolated nephropathy in two siblings from a Chinese pedigree. *Ren Fail*. 43:97-101.

Liu, Y.T., J. Hersheson, V. Plagnol, K. Fawcett, K.E. Duberley, E. Preza, I.P. Hargreaves, A. Chalasani, M. Laura, N.W. Wood, M.M. Reilly, and H. Houlden. 2014. Autosomal-recessive cerebellar ataxia caused by a novel ADCK3 mutation that elongates the protein: clinical, genetic and biochemical characterisation. *J Neurol Neurosurg Psychiatry*. 85:493-498.

Lolin, K., B.D. Chiodini, E. Hennaut, B. Adams, K. Dahan, and K. Ismaili. 2017. Early-onset of ADCK4 glomerulopathy with renal failure: a case report. *BMC Med Genet*. 18:28.

Lopez, L.C., M. Schuelke, C.M. Quinzii, T. Kanki, R.J. Rodenburg, A. Naini, S. Dimauro, and M. Hirano. 2006. Leigh syndrome with nephropathy and CoQ10 deficiency due to decaprenyl diphosphate synthase subunit 2 (PDSS2) mutations. *Am J Hum Genet*. 79:1125-1129.

Lu, M., Y. Zhou, Z. Wang, Z. Xia, J. Ren, and Q. Guo. 2019. Clinical phenotype, in silico and biomedical analyses, and intervention for an East Asian population-specific c.370G>A (p.G124S) COQ4 mutation in a Chinese family with CoQ10 deficiency-associated Leigh syndrome. *J Hum Genet*. 64:297-304.

Maeoka, Y., T. Doi, M. Aizawa, K. Miyasako, S. Hirashio, Y. Masuda, Y. Kishita, Y. Okazaki, K. Murayama, T. Imasawa, S. Hara, and T. Masaki. 2020. A case report of adult-onset COQ8B nephropathy presenting focal segmental glomerulosclerosis with granular swollen podocytes. *BMC Nephrol*. 21:376.

Malicdan, M.C.V., T. Vilboux, B. Ben-Zeev, J. Guo, A. Eliyahu, B. Pode-Shakked, A. Dori, S. Kakani, S.C. Chandrasekharappa, C.R. Ferreira, N. Shelestovich, D. Marek-Yagel, H. Pri-Chen, I. Blatt, J.E. Niederhuber, L. He, C. Toro, R.W. Taylor, J. Deeken, T. Yardeni, D.C. Wallace, W.A. Gahl, and Y. Anikster. 2018. A novel inborn error of the coenzyme Q10 biosynthesis pathway: cerebellar ataxia and static encephalomyopathy due to COQ5 C-methyltransferase deficiency. *Hum Mutat*. 39:69-79.

Mallaret, M., M. Renaud, C. Redin, N. Drouot, J. Muller, F. Severac, J.L. Mandel, W. Hamza, T. Benhassine, L. Ali-Pacha, M. Tazir, A. Durr, M.L. Monin, C. Mignot, P. Charles, L. Van Maldergem, L. Chamard, C. Thauvin-Robinet, V. Laugel, L. Burglen, P. Calvas, M.C. Fleury, C. Tranchant, M. Anheim, and M. Koenig. 2016. Validation of a clinical practice-based algorithm for the diagnosis of autosomal recessive cerebellar ataxias based on NGS identified cases. *Journal of neurology*. 263:1314-1322.

McCarthy, H.J., A. Bierzynska, M. Wherlock, M. Ognjanovic, L. Kerecuk, S. Hegde, S. Feather, R.D. Gilbert, L. Krischock, C. Jones, M.D. Sinha, N.J. Webb, M. Christian, M.M. Williams, S. Marks, A. Koziell, G.I. Welsh, M.A. Saleem, and R.t.U.S.S. Group. 2013. Simultaneous sequencing of 24 genes associated with steroid-resistant nephrotic syndrome. *Clin J Am Soc Nephrol*. 8:637-648.

Mero, S., L. Salviati, V. Leuzzi, A. Rubegni, C. Calderan, F. Nardecchia, D. Galatolo, M.A. Desbats, V. Naef, F. Gemignani, M. Novelli, A. Tessa, R. Battini, F.M. Santorelli, and M. Marchese. 2021. New pathogenic variants in COQ4 cause ataxia and neurodevelopmental disorder without detectable CoQ10 deficiency in muscle or skin fibroblasts. *Journal of neurology*. 268:3381-3389.

Mignot, C., E. Apartis, A. Durr, C. Marques Lourenco, P. Charles, D. Devos, C. Moreau, P. de Lonlay, N. Drouot, L. Burglen, N. Kempf, E. Nourisson, S. Chantot-Bastaraud, A.S. Lebre, M. Rio, Y. Chaix, E. Bieth, E. Roze, I. Bonnet, S. Canaple, C. Rastel, A. Brice, A. Rotig, I. Desguerre, C. Tranchant, M. Koenig, and M. Anheim. 2013. Phenotypic variability in ARCA2 and identification of a core ataxic phenotype with slow progression. *Orphanet J Rare Dis*. 8:173.

Mollet, J., A. Delahodde, V. Serre, D. Chretien, D. Schlemmer, A. Lombes, N. Boddaert, I. Desguerre, P. de Lonlay, H.O. de Baulny, A. Munnich, and A. Rotig. 2008. CABC1 gene mutations cause ubiquinone deficiency with cerebellar ataxia and seizures. *Am J Hum Genet*. 82:623-630.

Mollet, J., I. Giurgea, D. Schlemmer, G. Dallner, D. Chretien, A. Delahodde, D. Bacq, P. de Lonlay, A. Munnich, and A. Rotig. 2007. Prenyldiphosphate synthase, subunit 1 (PDSS1) and OH-benzoate polyprenyltransferase (COQ2) mutations in ubiquinone deficiency and oxidative phosphorylation disorders. *J Clin Invest*. 117:765-772.

Montini, G., C. Malaventura, and L. Salviati. 2008. Early coenzyme Q10 supplementation in primary coenzyme Q10 deficiency. *N Engl J Med*. 358:2849-2850.

Multiple-System Atrophy Research, C. 2013. Mutations in COQ2 in familial and sporadic multiple-system atrophy. *N Engl J Med*. 369:233-244.

Olgac, A., U. Oztoprak, C.S. Kasapkara, M. Kilic, D. Yuksel, E.B. Derinkuyu, Y. Tasci Yildiz, S. Ceylaner, and F.S. Ezgu. 2020. A rare case of primary coenzyme Q10 deficiency due to COQ9 mutation. *J Pediatr Endocrinol Metab*. 33:165-170.
Park, E., Y.H. Ahn, H.G. Kang, K.H. Yoo, N.H. Won, K.B. Lee, K.C. Moon, M.W. Seong, T.R. Gwon, S.S. Park, and H.I. Cheong. 2017a. COQ6 Mutations in Children With Steroid-Resistant Focal Segmental Glomerulosclerosis and Sensorineural Hearing Loss. *Am J Kidney Dis*. 70:139-144.
Park, E., H.G. Kang, Y.H. Choi, K.B. Lee, K.C. Moon, H.J. Jeong, M. Nagata, and H.I. Cheong. 2017b. Focal segmental glomerulosclerosis and medullary nephrocalcinosis in children with ADCK4 mutations. *Pediatr Nephrol*. 32:1547-1554.
Pineda, M., R. Montero, A. Aracil, M.M. O'Callaghan, A. Mas, C. Espinos, D. Martinez-Rubio, F. Palau, P. Navas, P. Briones, and R. Artuch. 2010. Coenzyme Q(10)-responsive ataxia: 2-year-treatment follow-up. *Movement disorders : official journal of the Movement Disorder Society*. 25:1262-1268.
Quinzii, C., A. Naini, L. Salviati, E. Trevisson, P. Navas, S. Dimauro, and M. Hirano. 2006. A mutation in para-hydroxybenzoate-polyprenyl transferase (COQ2) causes primary coenzyme Q10 deficiency. *Am J Hum Genet*. 78:345-349.
Quinzii, C.M., and M. Hirano. 2010. Coenzyme Q and mitochondrial disease. *Dev Disabil Res Rev*. 16:183-188.
Quinzii, C.M., L.C. Lopez, R.W. Gilkerson, B. Dorado, J. Coku, A.B. Naini, C. Lagier-Tourenne, M. Schuelke, L. Salviati, R. Carrozzo, F. Santorelli, S. Rahman, M. Tazir, M. Koenig, S. DiMauro, and M. Hirano. 2010. Reactive oxygen species, oxidative stress, and cell death correlate with level of CoQ10 deficiency. *FASEB journal : official publication of the Federation of American Societies for Experimental Biology*. 24:3733-3743.
Quinzii, C.M., L.C. Lopez, J. Von-Moltke, A. Naini, S. Krishna, M. Schuelke, L. Salviati, P. Navas, S. DiMauro, and M. Hirano. 2008. Respiratory chain dysfunction and oxidative stress correlate with severity of primary CoQ10 deficiency. *FASEB journal : official publication of the Federation of American Societies for Experimental Biology*. 22:1874-1885.
Rahman, S., I. Hargreaves, P. Clayton, and S. Heales. 2001. Neonatal presentation of coenzyme Q10 deficiency. *The Journal of pediatrics*. 139:456-458.
Romero-Moya, D., C. Santos-Ocana, J. Castano, G. Garrabou, J.A. Rodriguez-Gomez, V. Ruiz-Bonilla, C. Bueno, P. Gonzalez-Rodriguez, A. Giorgetti, E. Perdiguero, C. Prieto, C. Moren-Nunez, D.J. Fernandez-Ayala, M. Victoria Cascajo, I. Velasco, J.M. Canals, R. Montero, D. Yubero, C. Jou, J. Lopez-Barneo, F. Cardellach, P. Munoz-Canoves, R. Artuch, P. Navas, and P. Menendez. 2017. Genetic Rescue of Mitochondrial and Skeletal Muscle Impairment in an Induced Pluripotent Stem Cells Model of Coenzyme Q10 Deficiency. *Stem Cells*. 35:1687-1703.
Sadowski, C.E., S. Lovric, S. Ashraf, W.L. Pabst, H.Y. Gee, S. Kohl, S. Engelmann, V. Vega-Warner, H. Fang, J. Halbritter, M.J. Somers, W. Tan, S. Shril, I. Fessi, R.P. Lifton, D. Bockenhauer, S. El-Desoky, J.A. Kari, M. Zenker, M.J. Kemper, D. Mueller, H.M. Fathy, N.A. Soliman, S.S. Group, and F. Hildebrandt. 2015. A single-gene cause in 29.5% of cases of steroid-resistant nephrotic syndrome. *J Am Soc Nephrol*. 26:1279-1289.

Salviati, L., S. Sacconi, L. Murer, G. Zacchello, L. Franceschini, A.M. Laverda, G. Basso, C. Quinzii, C. Angelini, M. Hirano, A.B. Naini, P. Navas, S. DiMauro, and G. Montini. 2005. Infantile encephalomyopathy and nephropathy with CoQ10 deficiency: a CoQ10-responsive condition. *Neurology*. 65:606-608.

Salviati, L., E. Trevisson, M.A. Rodriguez Hernandez, A. Casarin, V. Pertegato, M. Doimo, M. Cassina, C. Agosto, M.A. Desbats, G. Sartori, S. Sacconi, L. Memo, O. Zuffardi, R. Artuch, C. Quinzii, S. Dimauro, M. Hirano, C. Santos-Ocana, and P. Navas. 2012. Haploinsufficiency of COQ4 causes coenzyme Q10 deficiency. *J Med Genet*. 49:187-191.

Scalais, E., R. Chafai, R. Van Coster, L. Bindl, C. Nuttin, C. Panagiotaraki, S. Seneca, W. Lissens, A. Ribes, C. Geers, J. Smet, and L. De Meirleir. 2013. Early myoclonic epilepsy, hypertrophic cardiomyopathy and subsequently a nephrotic syndrome in a patient with CoQ10 deficiency caused by mutations in para-hydroxybenzoate-polyprenyl transferase (COQ2). *Eur J Paediatr Neurol*. 17:625-630.

Schirinzi, T., M. Favetta, A. Romano, A. Sancesario, S. Summa, S. Minosse, G. Zanni, E. Castelli, E. Bertini, M. Petrarca, and G. Vasco. 2019. One-year outcome of coenzyme Q10 supplementation in ADCK3 ataxia (ARCA2). *Cerebellum Ataxias*. 6:15.

Sondheimer, N., S. Hewson, J.M. Cameron, G.R. Somers, J.D. Broadbent, M. Ziosi, C.M. Quinzii, and A.B. Naini. 2017. Novel recessive mutations in COQ4 cause severe infantile cardiomyopathy and encephalopathy associated with CoQ10 deficiency. *Mol Genet Metab Rep*. 12:23-27.

Stanczyk, M., I. Balasz-Chmielewska, B. Lipska-Zietkiewicz, and M. Tkaczyk. 2018. CoQ10-related sustained remission of proteinuria in a child with COQ6 glomerulopathy-a case report. *Pediatr Nephrol*. 33:2383-2387.

Sun, M., A.K. Johnson, V. Nelakuditi, L. Guidugli, D. Fischer, K. Arndt, L. Ma, E. Sandford, V. Shakkottai, K. Boycott, J. Warman-Chardon, Z. Li, D. Del Gaudio, M. Burmeister, C.M. Gomez, D.J. Waggoner, and S. Das. 2019. Targeted exome analysis identifies the genetic basis of disease in over 50% of patients with a wide range of ataxia-related phenotypes. *Genet Med*. 21:195-206.

Terracciano, A., F. Renaldo, G. Zanni, A. D'Amico, A. Pastore, S. Barresi, E.M. Valente, F. Piemonte, G. Tozzi, R. Carrozzo, M. Valeriani, R. Boldrini, E. Mercuri, F.M. Santorelli, and E. Bertini. 2012. The use of muscle biopsy in the diagnosis of undefined ataxia with cerebellar atrophy in children. *Eur J Paediatr Neurol*. 16:248-256.

Theunissen, T.E.J., M. Nguyen, R. Kamps, A.T. Hendrickx, S. Sallevelt, R.W.H. Gottschalk, C.M. Calis, A.P.M. Stassen, B. de Koning, E.N.M. Mulder-Den Hartog, K. Schoonderwoerd, S.A. Fuchs, Y. Hilhorst-Hofstee, M. de Visser, J. Vanoevelen, R. Szklarczyk, M. Gerards, I.F.M. de Coo, D. Hellebrekers, and H.J.M. Smeets. 2018. Whole Exome Sequencing Is the Preferred Strategy to Identify the Genetic Defect in Patients With a Probable or Possible Mitochondrial Cause. *Front Genet*. 9:400.

Traschutz, A., T. Schirinzi, L. Laugwitz, N.H. Murray, C.A. Bingman, S. Reich, J. Kern, A. Heinzmann, G. Vasco, E. Bertini, G. Zanni, A. Durr, S. Magri, F. Taroni, A. Malandrini, J. Baets, P. de Jonghe, W. de Ridder, M. Bereau, S. Demuth, C. Ganos, A.N. Basak, H. Hanagasi, S.H. Kurul, B. Bender, L. Schols, U. Grasshoff, T. Klopstock, R. Horvath, B. van de Warrenburg, L. Burglen, C. Rougeot, C. Ewenczyk, M. Koenig, F.M. Santorelli, M. Anheim, R.P. Munhoz, T. Haack, F. Distelmaier, D.J.

Pagliarini, H. Puccio, and M. Synofzik. 2020. Clinico-Genetic, Imaging and Molecular Delineation of COQ8A-Ataxia: A Multicenter Study of 59 Patients. *Annals of neurology*. 88:251-263.

Wang, F., Y. Zhang, J. Mao, Z. Yu, Z. Yi, L. Yu, J. Sun, X. Wei, F. Ding, H. Zhang, H. Xiao, Y. Yao, W. Tan, S. Lovric, J. Ding, and F. Hildebrandt. 2017a. Spectrum of mutations in Chinese children with steroid-resistant nephrotic syndrome. *Pediatr Nephrol*. 32:1181-1192.

Wang, N., Y. Zheng, L. Zhang, X. Tian, Y. Fang, M. Qi, J. Du, S. Chen, S. Chen, J. Li, B. Shen, and L. Wang. 2021. A Family Segregating Lethal Primary Coenzyme Q10 Deficiency Due to Two Novel COQ6 Variants. *Front Genet*. 12:811833.

Wang, Y., C. Smith, J.S. Parboosingh, A. Khan, M. Innes, and S. Hekimi. 2017b. Pathogenicity of two COQ7 mutations and responses to 2,4-dihydroxybenzoate bypass treatment. *J Cell Mol Med*.

Yang, J., Y. Yang, and Z. Hu. 2018. A novel ADCK4 mutation in a Chinese family with ADCK4-Associated glomerulopathy. *Biochem Biophys Res Commun*. 506:444-449.

Yu, M.H., M.H. Tsang, S. Lai, M.S. Ho, D.M.L. Tse, B. Willis, A.K. Kwong, Y.Y. Chou, S.P. Lin, C.M. Quinzii, W.L. Hwu, Y.H. Chien, P.L. Kuo, V.C. Chan, C. Tsoi, S.C. Chong, R.J.T. Rodenburg, J. Smeitink, C.C. Mak, K.S. Yeung, J.L. Fung, W. Lam, J. Hui, N.C. Lee, C.W. Fung, and B.H. Chung. 2019. Primary coenzyme Q10 deficiency-7: expanded phenotypic spectrum and a founder mutation in southern Chinese. *NPJ Genom Med*. 4:18.

Zhai, S.B., L. Zhang, B.C. Sun, Y. Zhang, and Q.S. Ma. 2020. Early-onset COQ8B (ADCK4) glomerulopathy in a child with isolated proteinuria: a case report and literature review. *BMC Nephrol*. 21:406.

Zhang, H., F. Wang, X. Liu, X. Zhong, Y. Yao, and H. Xiao. 2017. Steroid-resistant nephrotic syndrome caused by co-inheritance of mutations at NPHS1 and ADCK4 genes in two Chinese siblings. *Intractable Rare Dis Res*. 6:299-303.

Zhang, L., T. Ashizawa, and D. Peng. 2020. Primary coenzyme Q10 deficiency due to COQ8A gene mutations. *Mol Genet Genomic Med*. 8:e1420.

Ziosi, M., I. Di Meo, G. Kleiner, X.H. Gao, E. Barca, M.J. Sanchez-Quintero, S. Tadesse, H. Jiang, C. Qiao, R.J. Rodenburg, E. Scalais, M. Schuelke, B. Willard, M. Hatzoglou, V. Tiranti, and C.M. Quinzii. 2017. Coenzyme Q deficiency causes impairment of the sulfide oxidation pathway. *EMBO Mol Med*. 9:96-111.
